# Supplementary material for: Ecological genomics in the Northern krill uncovers loci for local adaptation across ocean basins
Source: Nat Commun. 2024 Aug 1;15:6297. doi: 10.1038/s41467-024-50239-7 (PMC11294593; doi:10.1038/s41467-024-50239-7)
Supplement: Supplementary file 1 — Supplementary Information [file 41467_2024_50239_MOESM1_ESM.pdf]

# Supplementary Information

## Ecological genomics in the Northern krill uncovers loci for local adaptation across ocean basins

Per Unneberg, Mårten Larsson, Anna Olsson, Ola Wallerman, Anna Petri, Ignas Bunikis, Olga Vinnere Pettersson, Chiara Papetti, Astthor Gislason, Henrik Glenner, Joan E. Cartes, Leocadio Blanco-Bercial, Elena Eriksen, Bettina Meyer, Andreas Wallberg*

*Corresponding author. Email: andreas.wallberg@imbim.uu.se (AW)

## Table of Contents


| Content | Page(s) |
|---|---|
| 1. Supplementary figures 1 – 26 | 2 – 36 |
| 2. Supplementary tables 1 – 5 | 37 – 41 |
| 3. Supplementary Note 1 | 42 |
| 4. Supplementary Note 2 | 43 – 45 |
| 5. References | 46 – 50 |

# Supplementary figures

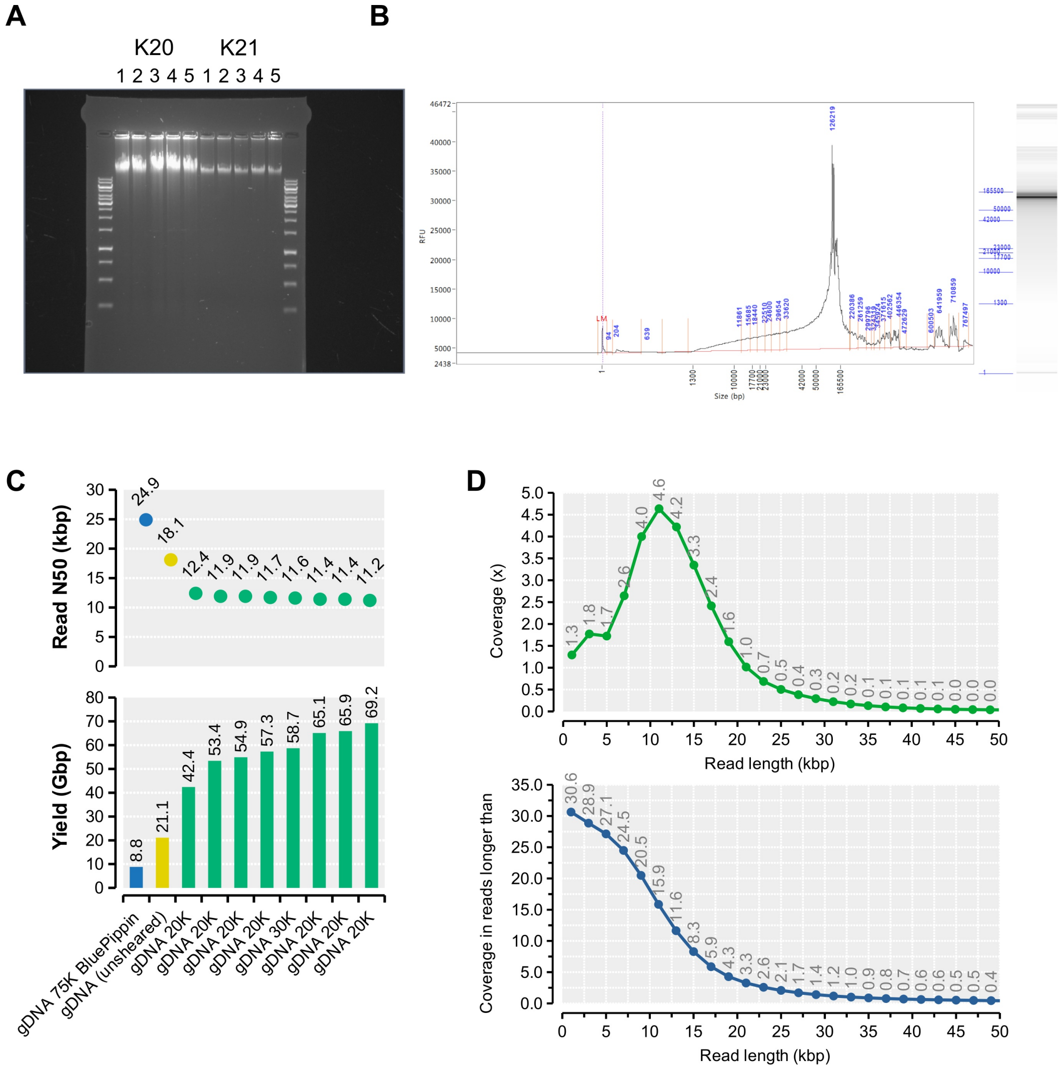


**Supplementary Figure 1:** Genomic Tip DNA extraction of the reference specimen sample K20 used for long-read and linked-read sequencing, as well as backup sample K21. (**A**) Agarose gel image of samples K20 and K21 (ladder: Thermo Scientific GeneRuler™1 kb DNA ladder). The gel image has a marginal crop at the bottom. (**B**) Femto Pulse fragment length readout of sample K20. (**C**) Nanopore flow cell yields using size-selected, unsheared or sheared DNA molecules. Shearing was done to fragments of 20 or 30 kb. (**D**) Genome coverage for PromethION reads of different length intervals. Top=genome coverage for a particular length class. Bottom=genome coverage for a particular length class or longer reads.

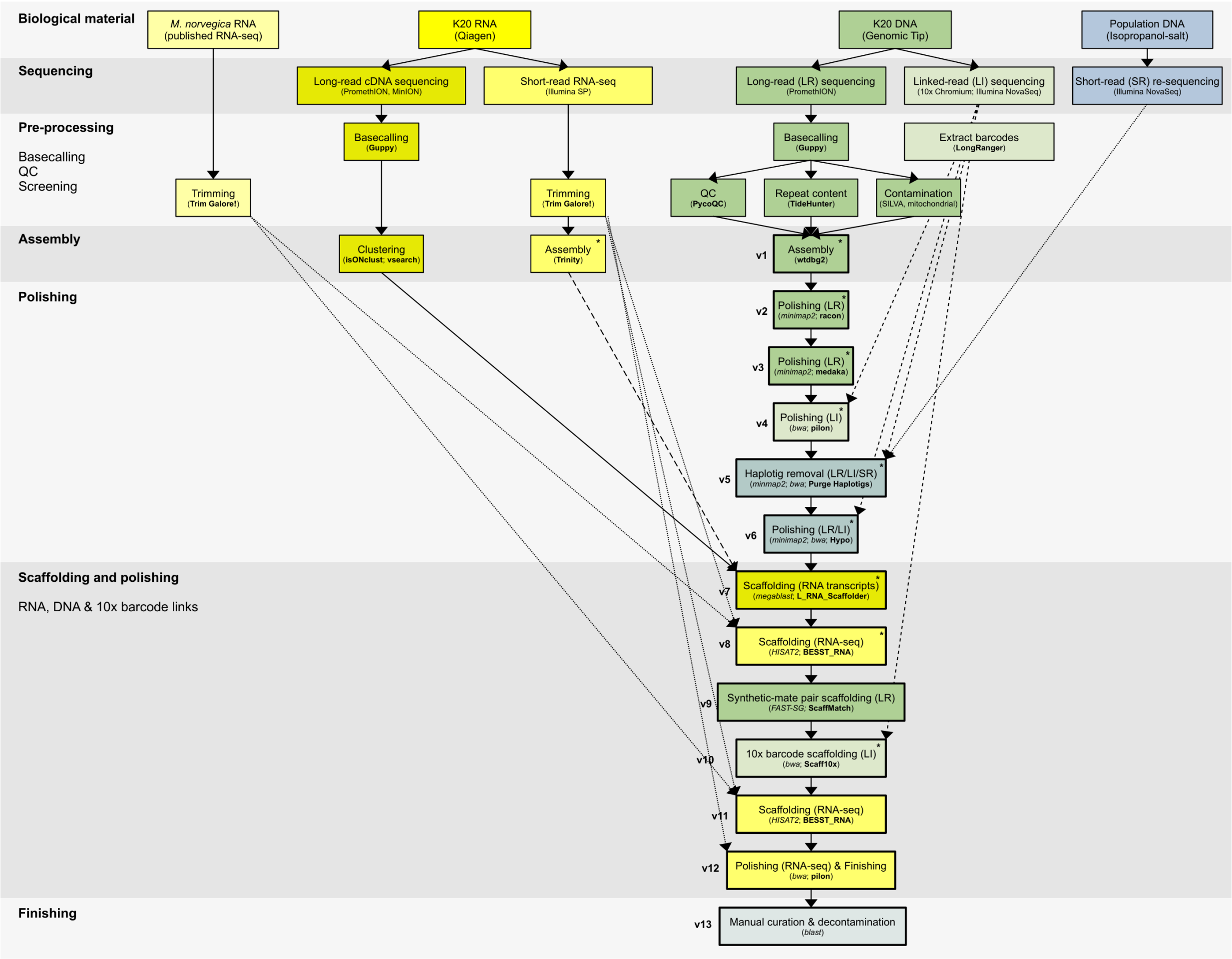


**Supplementary Figure 2:** Genome assembly workflow overview. Biological material included RNA data from published samples, RNA and DNA data from the reference specimen "K20" and population resequencing data. For the first two steps (Biological materials and Sequencing), extraction and sequencing methods are indicated in parentheses, while main bioinformatics tools are indicated in subsequent steps. Pre-processing of data encompassed basecalling, trimming, processing of barcodes and basic analytics including quality control (QC) and screening for contaminants (Screening). Colors of boxes indicate important data types: yellow=data and steps involving RNA (e.g. scaffolding and annotation); green=data and steps reliant on DNA derived from the reference specimen (e.g. assembly and polishing); light blue=population data. Arrows indicate how the RNA and DNA materials were used throughout the genome assembly workflow. A final round of manual curation and removal of putative bacterial contaminants generated assembly version 13.

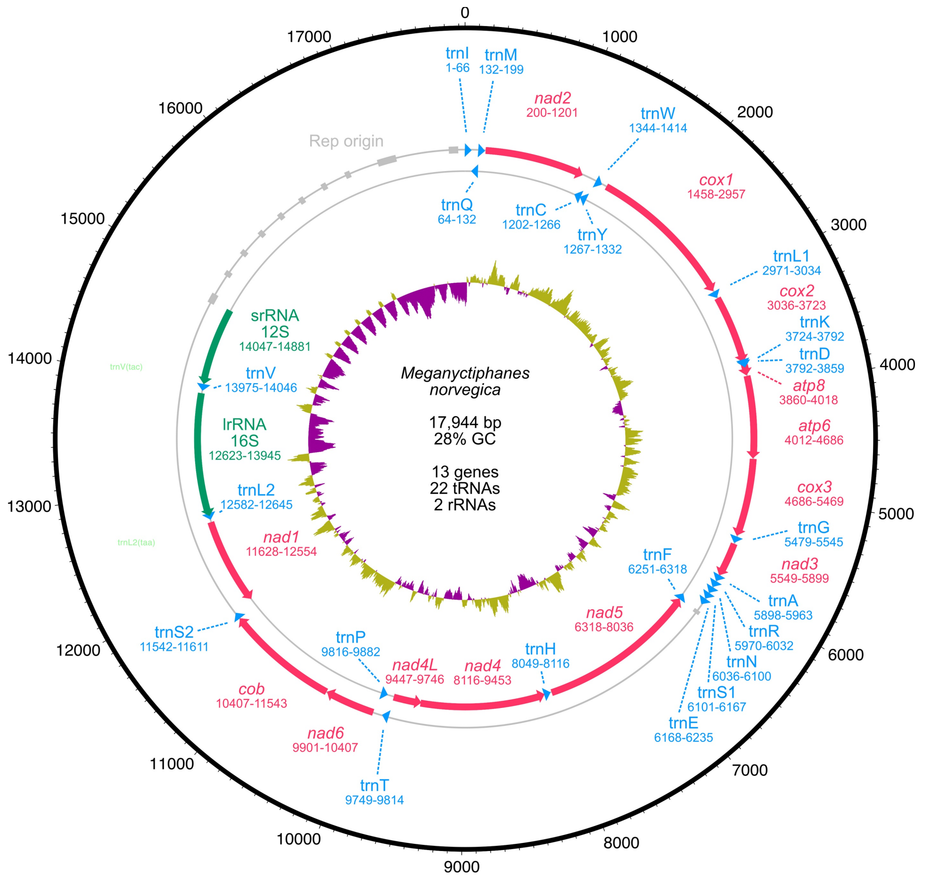


**Supplementary Figure 3:** A 17 kbp mitochondrial chromosome assembly and features derived from PromethION long-reads from the K20 reference specimen. Coordinates are indicated on the outer track (bold black line). Inner histogram indicates GC-content (pink= %GC below mean; green=%GC above mean). Detected features are indicated on the outer heavy/plus strand (clockwise) or the light/minus strand (counter-clockwise), respectively: red arrows=protein coding genes; green arrows=ribosomal RNAs (rRNA); blue arrows=transfer RNA (tRNA); gray arrows=origin of replication and/or AT-rich repeats). Summary statistics are indicated at the center of the plot.

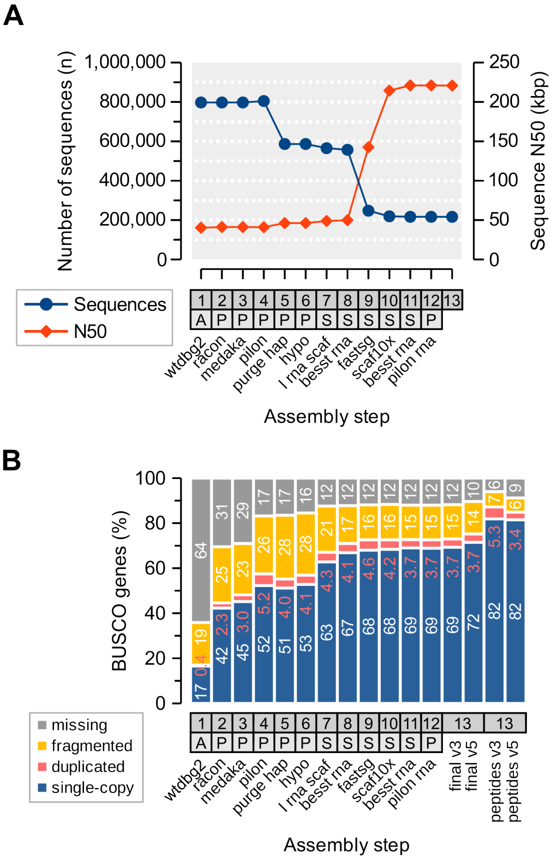


**Supplementary Figure 4:** Genome assembly metrics. (**A**) Number and N50 lengths of genome sequences throughout the assembly pipeline. Steps 1–12 involved bioinformatic tasks (A=assembly; P=polishing; S=scaffolding) while step 13 was a mix of manual curation and bioinformatics to accomplish the final assembly. (**B**) BUSCO gene detection analysis using the Arthropod odb9 gene set and BUSCO v3 in genome mode (steps 1–12) or both BUSCOv3/odb9 and BUSCOv5/odb10 in both genome mode or peptide mode.

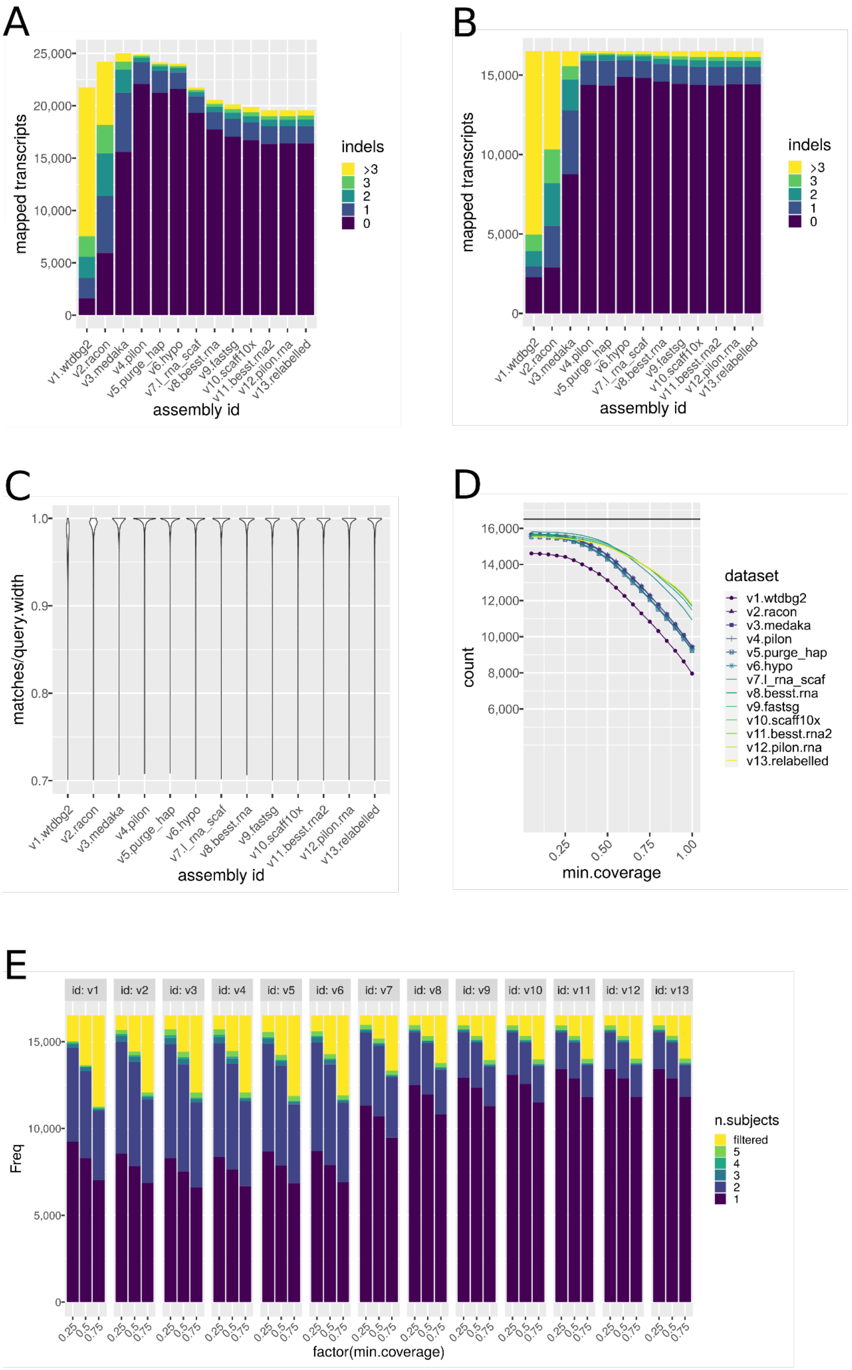


**Supplementary Figure 5:** Genome assembly metrics based on mapping properties of Trinity transcripts (n=16,509 input sequences). Distribution of insertion-deletions (indels) over mapped transcripts (**A-B**) for redundant mappings (**A**) and averaged over each transcript (**B**). The numbers of indels decrease rapidly for the first rounds of polishing and thereafter remain stable during the subsequent scaffolding steps. Similarly, the number of mismatches decreases as a result of polishing (**C**). The number of transcripts mapping to multiple contigs decreases as a result of scaffolding (**D**), indicating an improvement in assembly contiguity. Assembly completeness is assessed by looking at the number of gene bodies covered to a certain extent at 75% identity (**E**).

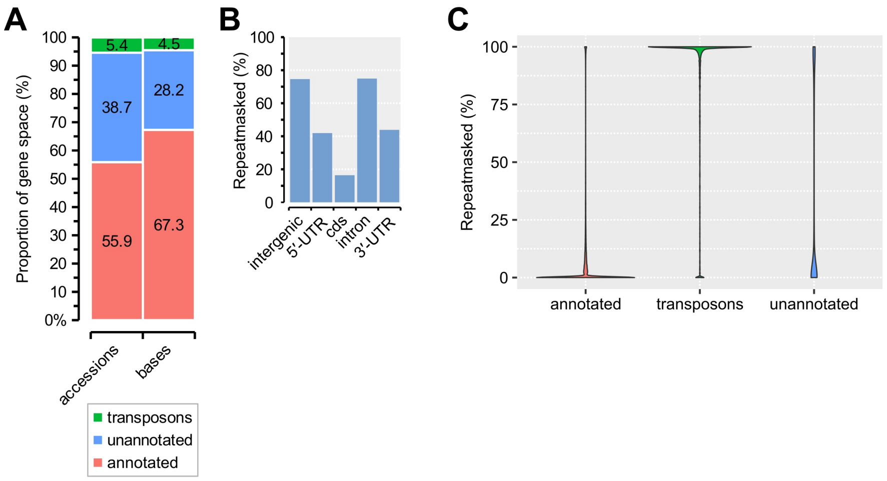


**Supplementary Figure 6:** Overview of 42,227 putative protein-coding gene models in the krill genome detected through RNA and comparative data and functionally annotated using homology information in EnTAP. (**A**) The proportion of regular genes ("annotated"; n=25,301), transposons (n=2,283) and unannotated genes (n=14,643) following functional analysis with EnTAP and searches for transposon keywords ("accessions"=proportion of the total number of all genes; "bases"=proportion of the total number of bases of the coding sequence of all genes). (**B**) The genome-average repeat content estimated for each genomic region (UTR=untranslated exonic regions; cds=coding sequence) estimated with a custom library of interspersed repeats using RepeatMasker. For example, about ~15% of the coding sequence appears repetitive. (**C**) The per-gene distribution of repeat content across the 42,227 gene models. Categories as in (A). The bimodal repeat-content among unannotated genes hints at thousands of additional genes and TEs.

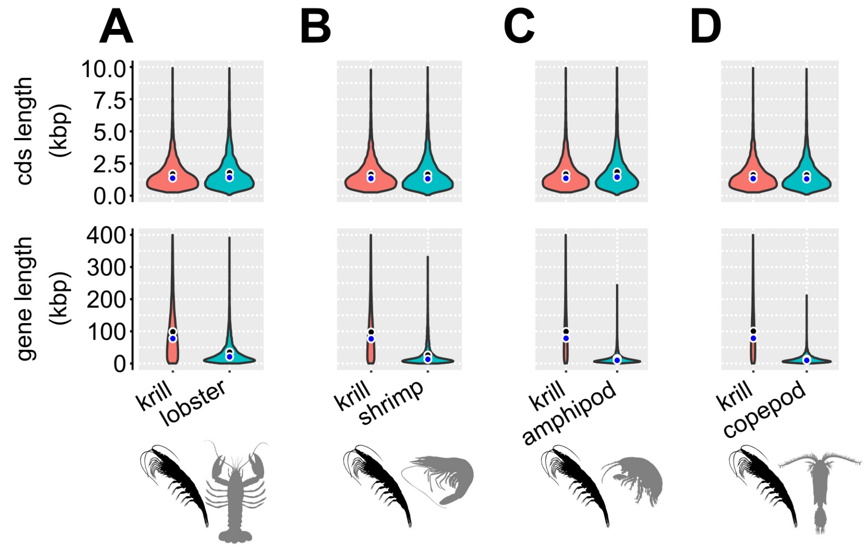


**Supplementary Figure 7:** Comparison of the lengths of genes and coding sequences of 1:1 orthologs between *M. norvegica* and other crustacean species. (**A**) Comparison between *M. norvegica* vs. American lobster *H. americanus* based on 7,150 1:1 orthologs. Upper plot: distribution of the lengths of coding sequences (cds). Black circle indicates mean cds and blue circle indicates median cds. Lower plot: lengths of the full gene bodies in each genome. Mean and medians as in the upper plot. (**B**) Comparison between *M. norvegica* vs. Black tiger shrimp *P. monodon* based on 7,084 1:1 orthologs. Plots and scales as in (A). (**C**) Comparison between *M. norvegica* vs. the amphipod *H. azteca* based on 5,836 1:1 orthologs. Plots and scales as in (A). (**D**) Comparison between *M. norvegica* vs. the copepod *E. affinis* based on 4,551 1:1 orthologs. Plots and scales as in (A).

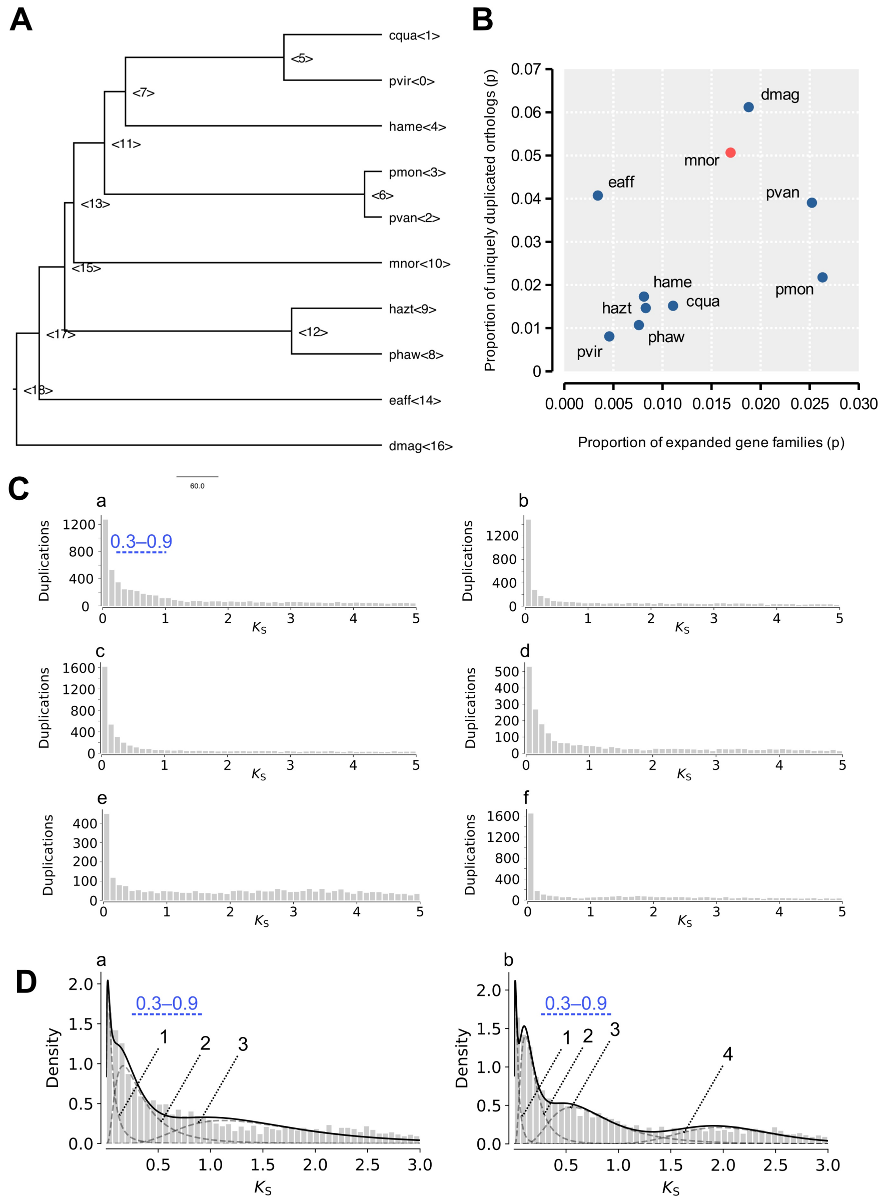


**Supplementary Figure 8:** Gene family expansion, gene duplication rates and synonymous (presumably neutral) divergence among gene family paralogs across crustaceans. (**A**) Phylogenetic time-tree used in gene family expansion analyses in CAFE. Node and tip labels are given in brackets and match those in Supplementary Table 11. Labels: mnor=*Meganyctiphanes norvegica*; hame=*Homarus americanus*; cqua=*Cherax quadricarinatus*; pvir=*Procambarus virginalis*; pmon=*Penaeus monodon*; pvan=*Penaeus vannamei*; hazt=*Hyalella azteca*; phaw=*Parhyale hawaiensis*; Eaff=*Eurytemora affinis*; Dmag=*Daphnia magna*. (**B**) Dot plot of lineage-specific duplicated genes and rapidly evolving gene families. X-axis: the rate of gene family expansion, measured as the proportion of rapidly evolving gene families relative to all gene families in that species and inferred through phylogenetic analysis in CAFE ($p<0.05$ that these families are evolving at the same rate as the genome-wide average background rate). Y-axis: proportion of lineage-specific expanded gene families for which orthologs are otherwise only found as single-copy genes in other species (allowing for two species with missing data). Labels as in A. (**C**) The distribution of synonymous divergence ($K_S$) among gene family paralogs in six species using node-weighted calculations in gene family trees. Sub-panels are: a) *M. norvegica*; b) *H. americanus*; c) *P. monodon*; d) *H. azteca*; e) *E. affinis*; f) *D. magna*. A potential peak or shoulder within the $K_S$-range from 0.3 to 0.9 in the krill is highlighted in blue. (**D**) Results of

fitting Gaussian mixture models (GMMs) to the observed $K_S$-distribution in the krill using either three components (a) or four components (b) visualized with dashed and dotted lines (components are indicated by numbers).

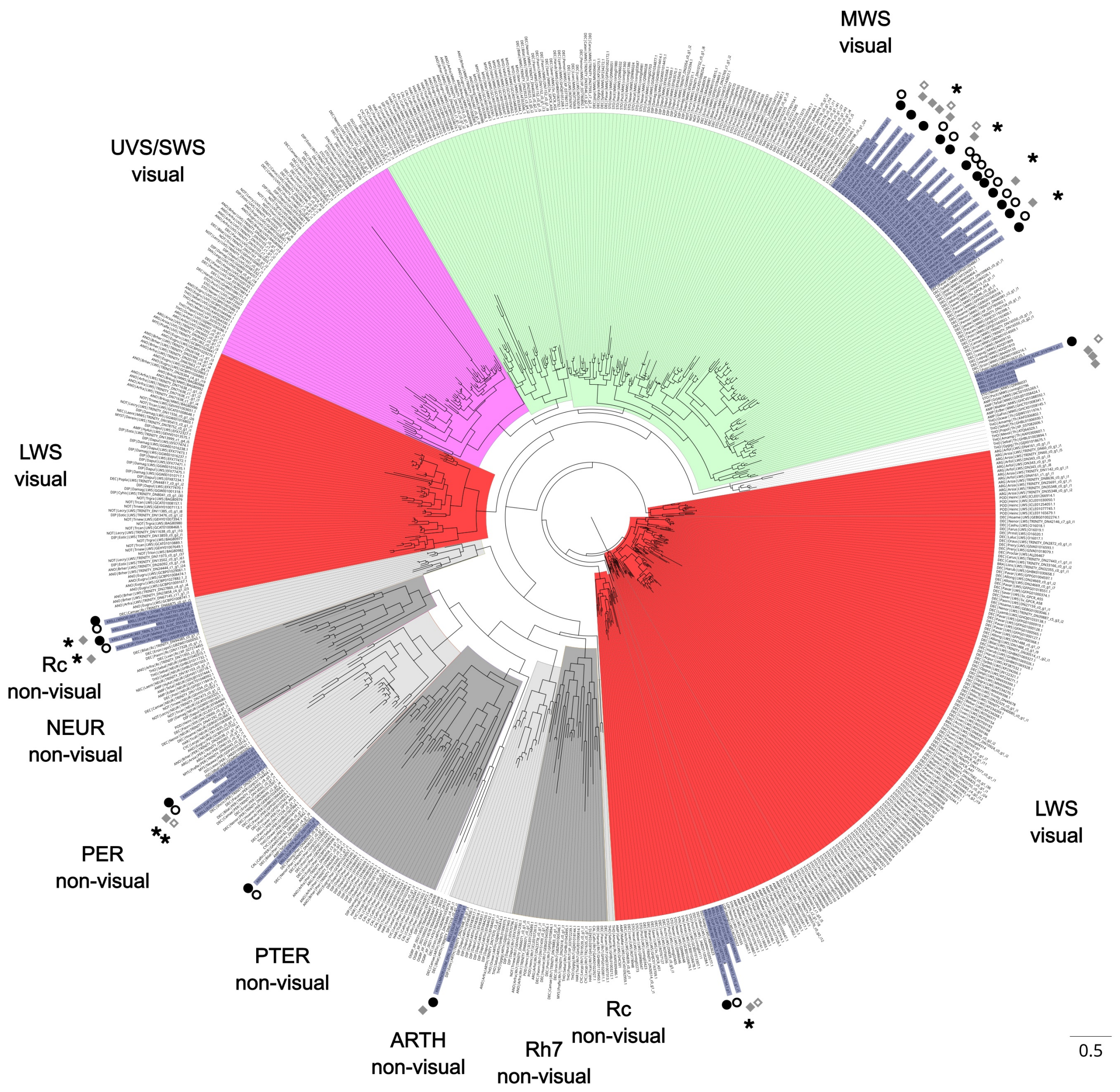


**Supplementary Figure 9:** A maximum-likelihood tree of crustacean opsin gene family proteins (n=663 sequences). Genes from the Northern krill *M. norvegica* genome assembly (n=19) and transcripts from Antarctic krill *E. superba* (n=15) from Urso *et al.*[1] were added to the crustacean dataset of Palecanda *et al.* [2]. Major opsin groups including visual or non-visual function indicated are: LWS=long wavelength-sensitive; MWS=middle wavelength-sensitive; UVS=ultraviolet/short wavelength-sensitive; Rc=crustaceanrhabdomeric opsin; Rh7=rhodopsin 7; ARTH=arthropsins; NEUR=neuropsins; PTER=pteropsins; PER=peropsins. Black circles=*M. norvegica* genes; White circles=*M. norvegica* transcripts from Palecanda *et al.*; Grey diamonds=*E. superba* transcripts from Urso *et al.*; White diamonds=*E. superba* markers from Palecanda *et al.*; stars=*Thysanoessa inermis* krill transcripts from Palecanda *et al.*

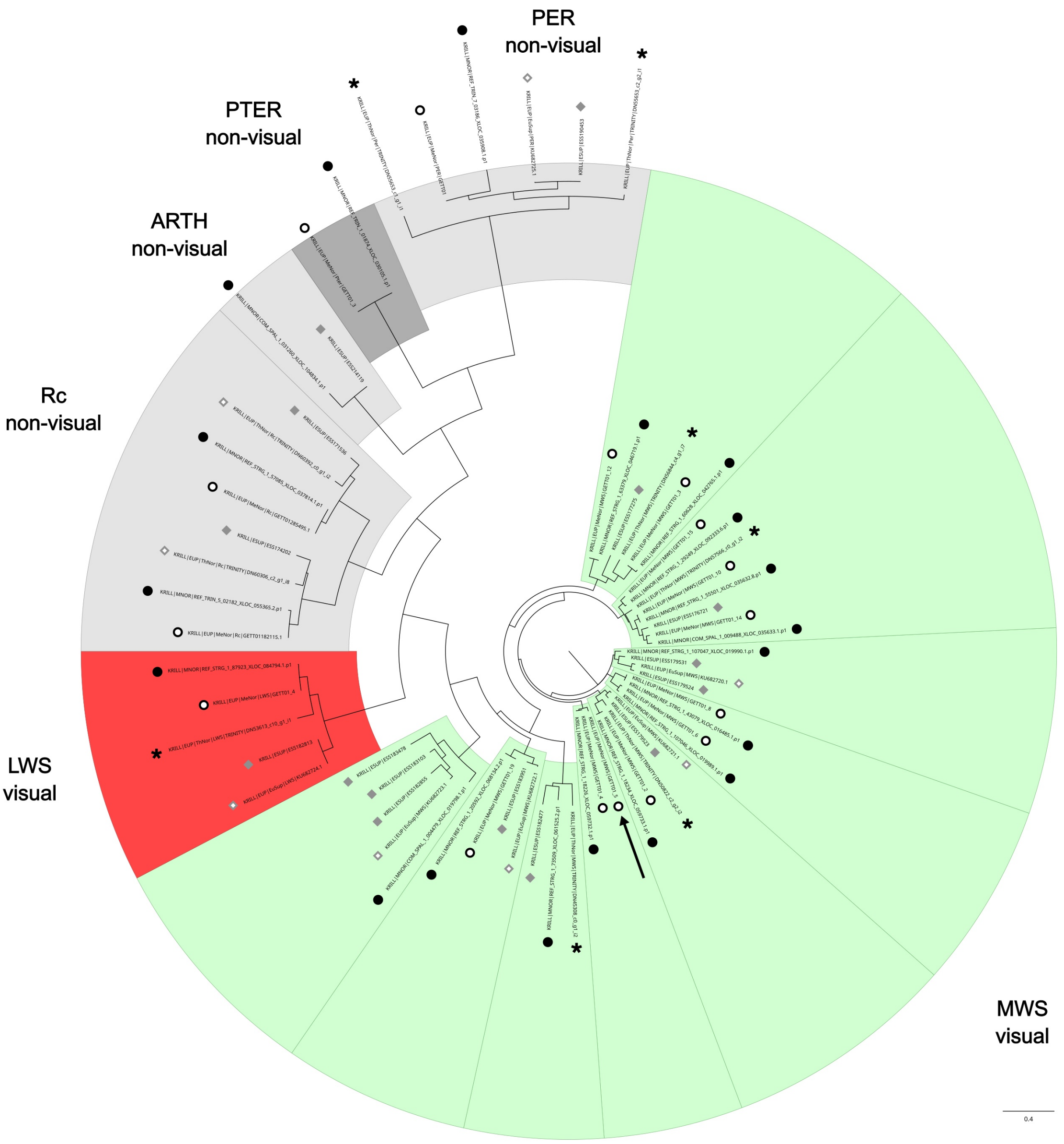


**Supplementary Figure 10:** A maximum-likelihood tree of opsin gene family proteins in the krill (n=65 sequences). Genes from the Northern krill *M. norvegica* genome assembly and transcripts from Antarctic krill *E. superba* from Urso *et al.*[1] were added to the krill subset of the dataset from Palecanda *et al.*[2]. Major opsin groups including visual or non-visual function indicated are: LWS=long wavelength-sensitive; MWS=middle wavelength-sensitive; Rc=crustaceanrhabdomeric opsin; ARTH=arthropsins; PTER=pteropsins; PER=peropsins. Black circles=*M. norvegica* genes; White circles=*M. norvegica* transcripts from Palecanda *et al.*; Grey diamonds=*E. superba* transcripts from Urso *et al.*; White diamonds=*E. superba* markers from Palecanda *et al.*; stars=*Thysanoessa inermis* krill transcripts from Palecanda *et al.* Arrow indicates an *M. norvegica* RNA transcript that is not unambiguously paired with a gene in the *M. norvegica* genome assembly.

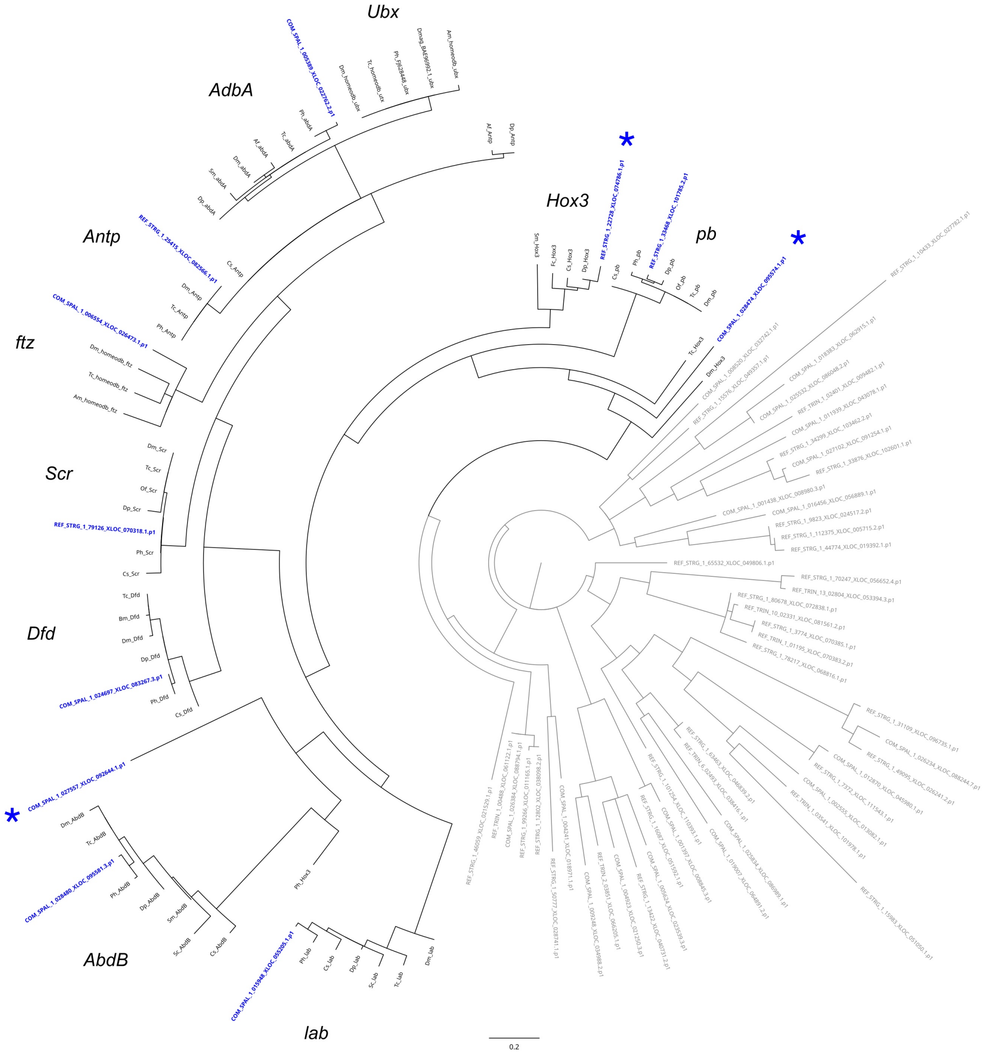


**Supplementary Figure 11:** Phylogenetic inference of Hox genes in the Northern krill genome. A maximum likelihood phylogenetic tree was inferred from homeodomain motifs of 10 core Hox genes in arthropods (crustaceans+insects) taxa together with homeodomain gene candidates in *M. norvegica* (n=119 sequences in total). Blue labels indicate Northern krill accessions that fall within the Hox gene clade and respective Hox-gene branches. Non-hox *M. norvegica* accessions in gray. Genes: *lab=labial*; *pb=proboscipedia*; *Hox3*, *Dfd=Deformed*; *Scr=Sex combs reduced*; *ftz=fushi tarazu*, *Antp=Antennapedia*; *Abd-A=abdominal-A*; *Abd-B=Abdominal-B*; *Ubx=Ultrabithorax*. Nine out ten Hox genes were detected in krill, eight of which as single-copy genes. Three putative *Hox3*-like paralogs are indicated with stars. Species: Ag=*Anopheles gambiae*; Af=*Artemia franciscana*; Am=*Apis mellifera*; Bm=*Bombyx mori*; Cs=*Cupiennius salei*; Dp=*Daphnia pulex*; Dm=*Drosophila melanogaster*; Dmag=*Daphnia magna*; Of=*Oncopeltus fasciatus*; Ph=*Parhyale hawaiensis*; Ps=*Porcellio scaber*; Pc=*Procambarus clarkii*; Sc=*Sacculina carcini*; Sm=*Strigamia maritima*; Td=*Thermobia domestica*; Tc=*Tribolium castaneum*.

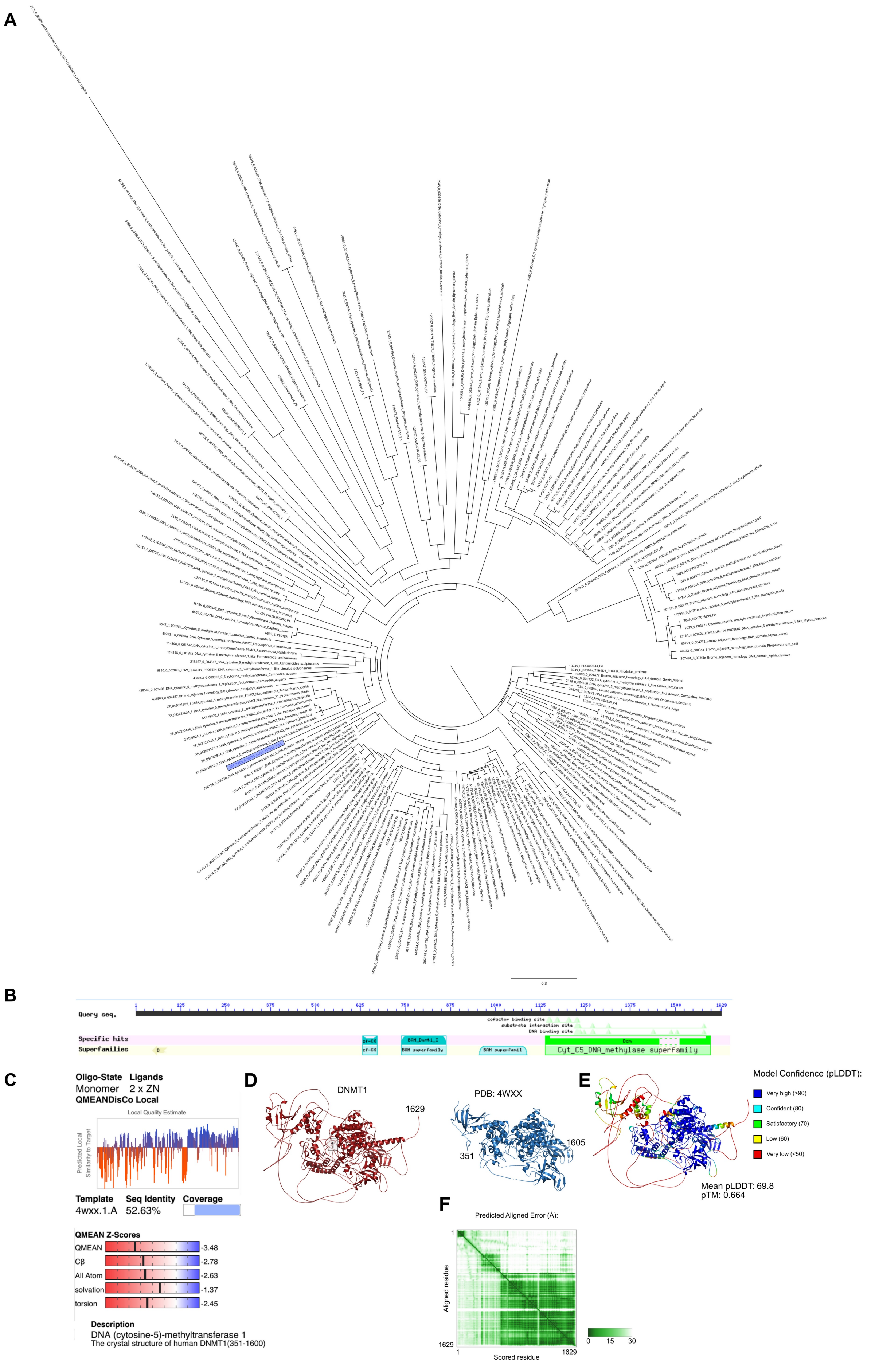
A
B
Query seq.
cofactor binding site
substrate interaction site
DNA binding site
Specific hits
Superfamilies
zf-CX
BAH_Dnmt1_I
BAH superfamily
BAH superfamil
Dcm
Cyt_C5_DNA_methylase superfamily
C
Oligo-State Ligands
Monomer 2 x ZN
QMEANDisCo Local
Local Quality Estimate
Predicted Local Similarity to Target
Template Seq Identity Coverage
4wxx.1.A 52.63%
QMEAN Z-Scores
QMEAN -3.48
Cβ -2.78
All Atom -2.63
solvation -1.37
torsion -2.45
Description
DNA (cytosine-5)-methyltransferase 1
The crystal structure of human DNMT1(351-1600)
D
DNMT1
1629
PDB: 4WXX
351
1605
E
Model Confidence (pLDDT):
Very high (>90)
Confident (80)
Satisfactory (70)
Low (60)
Very low (<50)
Mean pLDDT: 69.8
pTM: 0.664
F
Predicted Aligned Error (Å):
Aligned residue
Scored residue
0 15 30

**Supplementary Figure 12:** Comparative and functional analysis of a candidate gene encoding DNMT1 in *M. norvegica* (REF_TRIN_3_00283_XLOC_111019.1.p1). The gene candidate was first detected in the EnTAP annotations and then analyzed for arthropod homologs. (**A**) Phylogenetic analysis using orthologs from OrthoDB, EggNOG and NCBI. Blue label indicates the position of the candidate krill sequence. See Supplementary Data 7 for numbers and labels of sequences (n=197 sequences in total). (**B**) Domains indicated from querying the peptide sequence against the NCBI Conserved Domains database. (**C**) The best homology hit (model 4wxx.1.A) from querying the peptide sequence against the SWISS-MODEL database. (**D–F**) Modeling of the DNMT1 protein. (**D**) Comparison of the ColabFold predicted DNMT1 protein model (red) to the crystal structure of the human DNMT1 (PDB: 4WXX, blue)[3]. (**E**) Per-residue confidence coloring of the top ranked predicted model of DNMT1. The mean predicted local distance difference test (pLDDT) value and pTM score are annotated. (**F**) Residue-residue alignment plot of the predicted DNMT1 model.

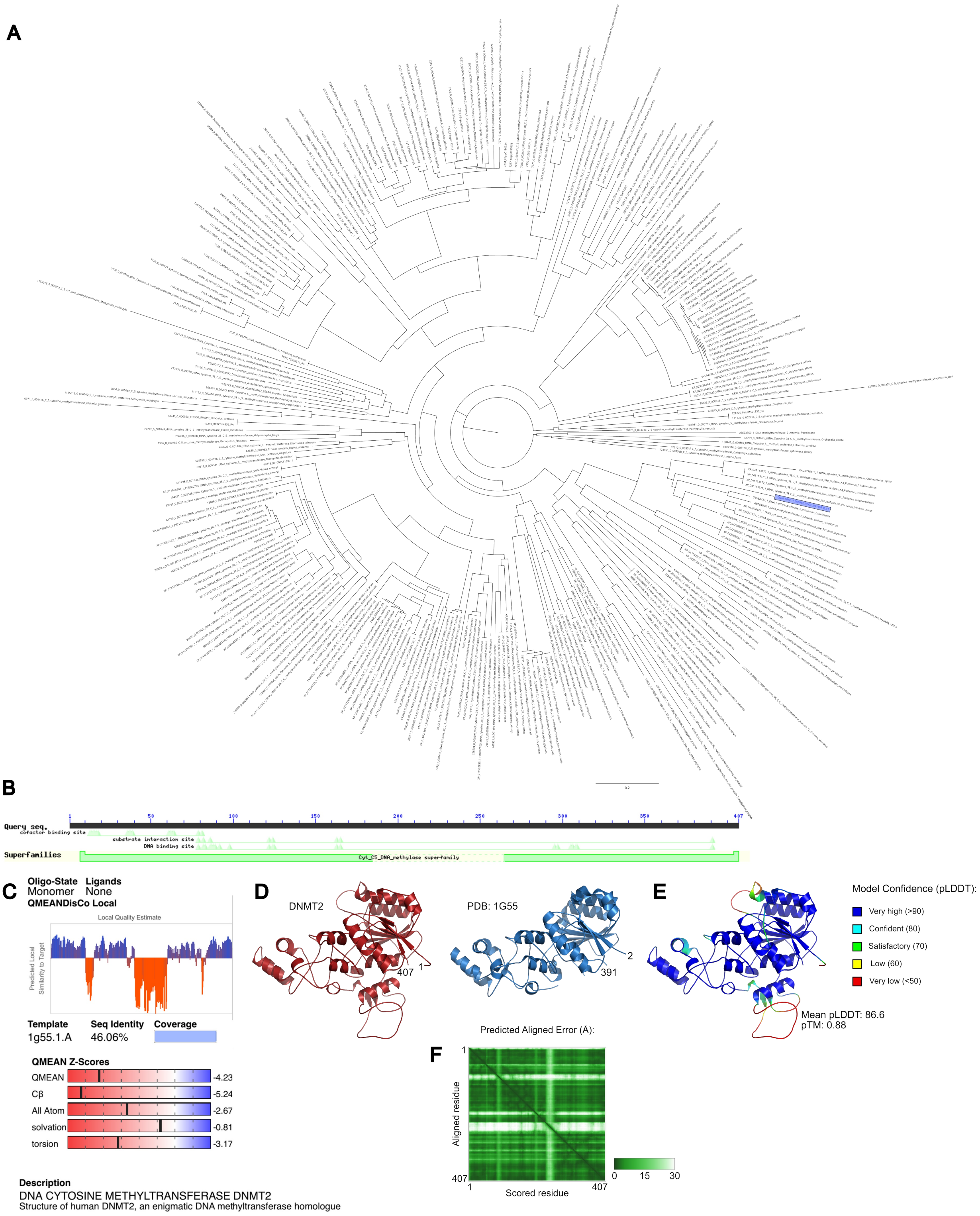


**Supplementary Figure 13:** Comparative and functional analysis of a candidate gene encoding DNMT2 in *M. norvegica* (COM_SPAL_1_008092_XLOC_031468.8.p1). The gene candidate was first detected in the EnTAP annotations and then analyzed against arthropod homologs. (**A**) Phylogenetic analysis using orthologs from OrthoDB, EggNOG and NCBI. Blue label indicates the position of the candidate krill sequence. See Supplementary Data 7 for numbers and labels of sequences (n=305 sequences in total). (**B**) Domains indicated from querying the peptide sequence against the NCBI Conserved Domains database. (**C**) The best

homology hit (model 1g55.1.A) from querying the peptide sequence against the SWISS-MODEL database. (**D–F**) Modeling of the DNMT2 protein. (**D**) Comparison of the ColabFold predicted DNMT2 protein model (red) to the crystal structure of the human DNMT2 (PDB: 1G55, blue)[4]. (**E**) Per-residue confidence coloring of the top ranked predicted model of DNMT2. The mean predicted local distance difference test (pLDDT) value and pTM score are annotated. (**F**) Residue-residue alignment plot of the predicted DNMT2 model.

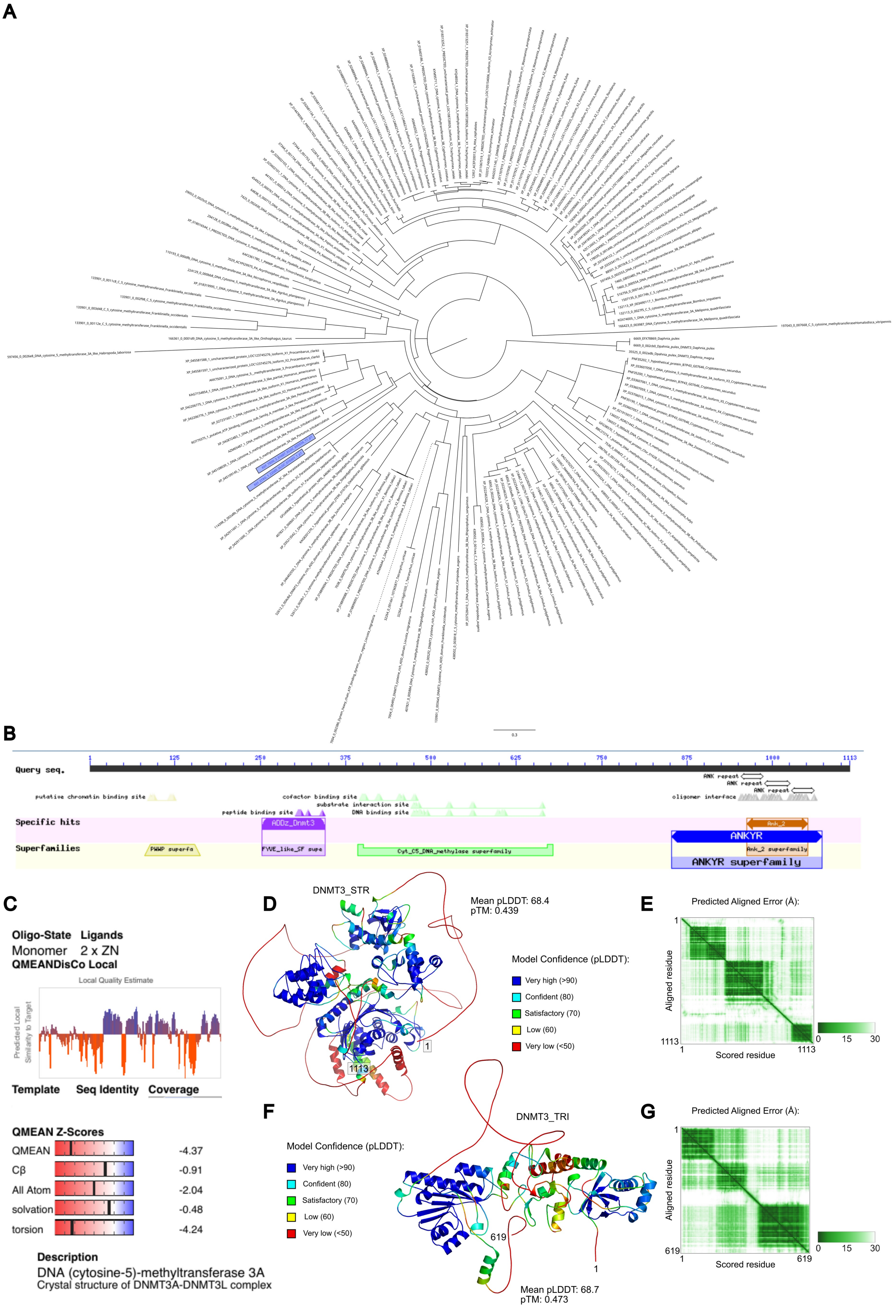


**Supplementary Figure 14:** Comparative and functional analysis of two candidate genes encoding DNMT3 homologs in *M. norvegica* (REF_TRIN_3_01687_XLOC_055556_11.p1

and REF_STRG_1_22585_XLOC_074301_1.p1). The gene candidates were first detected in the EnTAP annotations and then analyzed against arthropod homologs. (**A**) Phylogenetic analysis using orthologs from OrthoDB, EggNOG and NCBI. One dotted branch has been shortened for presentation. Blue label indicates the position of the candidate krill sequence. See Supplementary Data 7 for numbers and labels of sequences (n=156 sequences in total). (**B**) Domains indicated from querying the REF_STRG_1_22585_XLOC_074301_1.p1 peptide sequence against the NCBI Conserved Domains database. (**C**) The best homology hit (model 4u7p.1.A) from querying the peptide sequence REF_STRG_1_22585_XLOC_074301_1.p1 against the SWISS-MODEL database. (**D–F**) Modeling of the REF_STRG_1_22585_XLOC_074301_1 protein. (**D**) Per-residue confidence coloring of the top ranked predicted model of DNMT3_STR. The mean predicted local distance difference test (pLDDT) value and pTM score are annotated. (**E**) Residue-residue alignment plot of the predicted DNMT3_STR model. (**F–G**) Modeling of the REF_TRIN_3_01687_XLOC_055556_11 protein. (**F**) Per-residue confidence coloring of the top ranked predicted model of DNMT3_TRI. The mean predicted local distance difference test (pLDDT) value and pTM score are annotated. (**G**) Residue-residue alignment plot of the predicted DNMT3_TRI model.

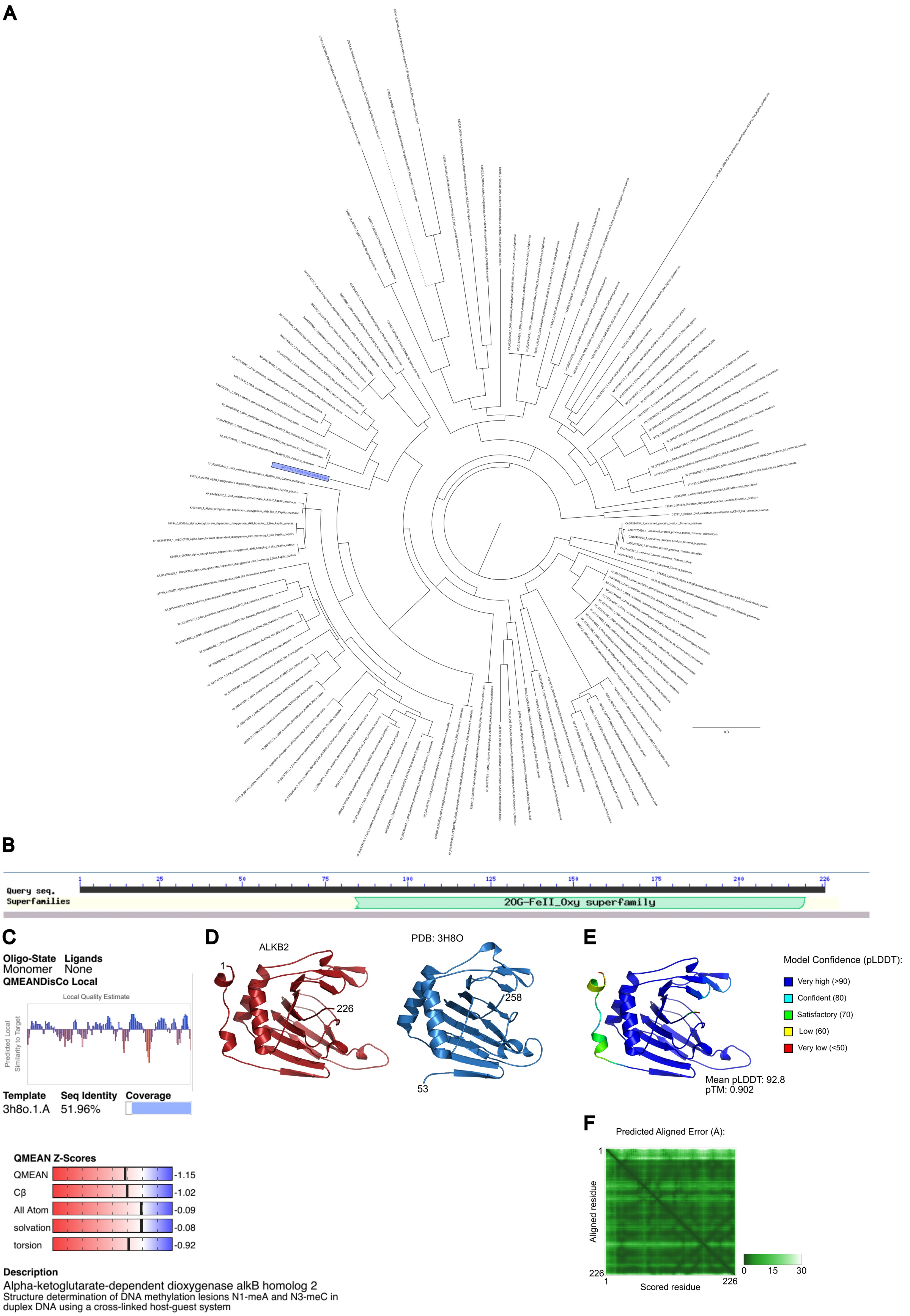


**Supplementary Figure 15:** Comparative and functional analysis of a candidate gene encoding ALKB2 in *M. norvegica* (COM_SPAL_1_008092_XLOC_031468.8.p1). The gene

candidate was first detected in the EnTAP annotations and then analyzed against arthropod homologs. (**A**) Phylogenetic analysis using orthologs from OrthoDB and NCBI. Blue label indicates the position of the candidate krill sequence. See Supplementary Data 7 for numbers and labels of sequences (n=124 sequences in total). (**B**) Domains indicated from querying the peptide sequence against the NCBI Conserved Domains database. (**C**) The best homology hit (model 3h8o.1.A) from querying the peptide sequence against the SWISS-MODEL database. (**D–F**) Modeling of the ALKB2 protein. (**D**) Comparison of the ColabFold predicted ALKB2 protein model (red) to the crystal structure of the human ALKB2 (PDB: 3H8O, blue)[5]. (**E**) Per-residue confidence coloring of the top ranked predicted model of ALKB2. The mean predicted local distance difference test (pLDDT) value and pTM score are annotated. (**F**) Residue-residue alignment plot of the predicted ALKB2 model.

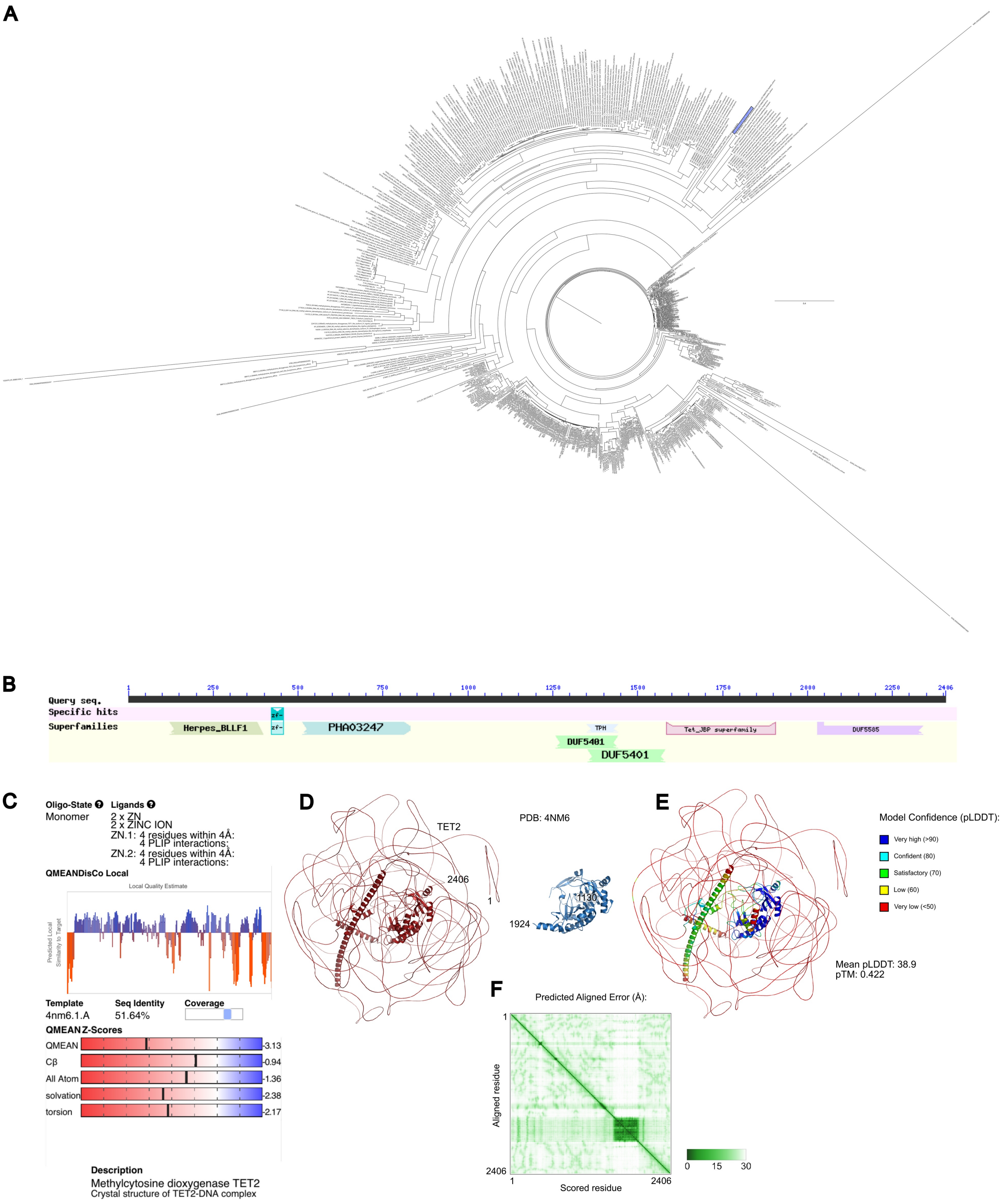


**Supplementary Figure 16:** Comparative and functional analysis of a candidate gene encoding TET2 in *M. norvegica* (REF_STRG_1_37441_XLOC_109451.1.p1). The gene candidates were first detected in the EnTAP annotations and then analyzed against arthropod homologs. (**A**) Phylogenetic analysis using orthologs from OrthoDB and EggNOG. Blue label indicates the position of the candidate krill sequence. See Supplementary Data 7 for numbers and labels of sequences (n=594 sequences in total). (**B**) Domains indicated from querying the peptide sequence against the NCBI Conserved Domains database. (**C**) The best homology hit (model 4nm6.1.A) from querying the peptide sequence against the SWISS-MODEL database. (**D–F**) Modeling of the TET2 protein. (**D**) Comparison of the ColabFold

predicted TET2 protein model (red) to the crystal structure of the human TET2 (PDB: 4NM6, blue)[6]. (**E**) Per-residue confidence coloring of the top ranked predicted model of TET2. The mean predicted local distance difference test (pLDDT) value and pTM score are annotated. (**F**) Residue-residue alignment plot of the predicted TET2 model.

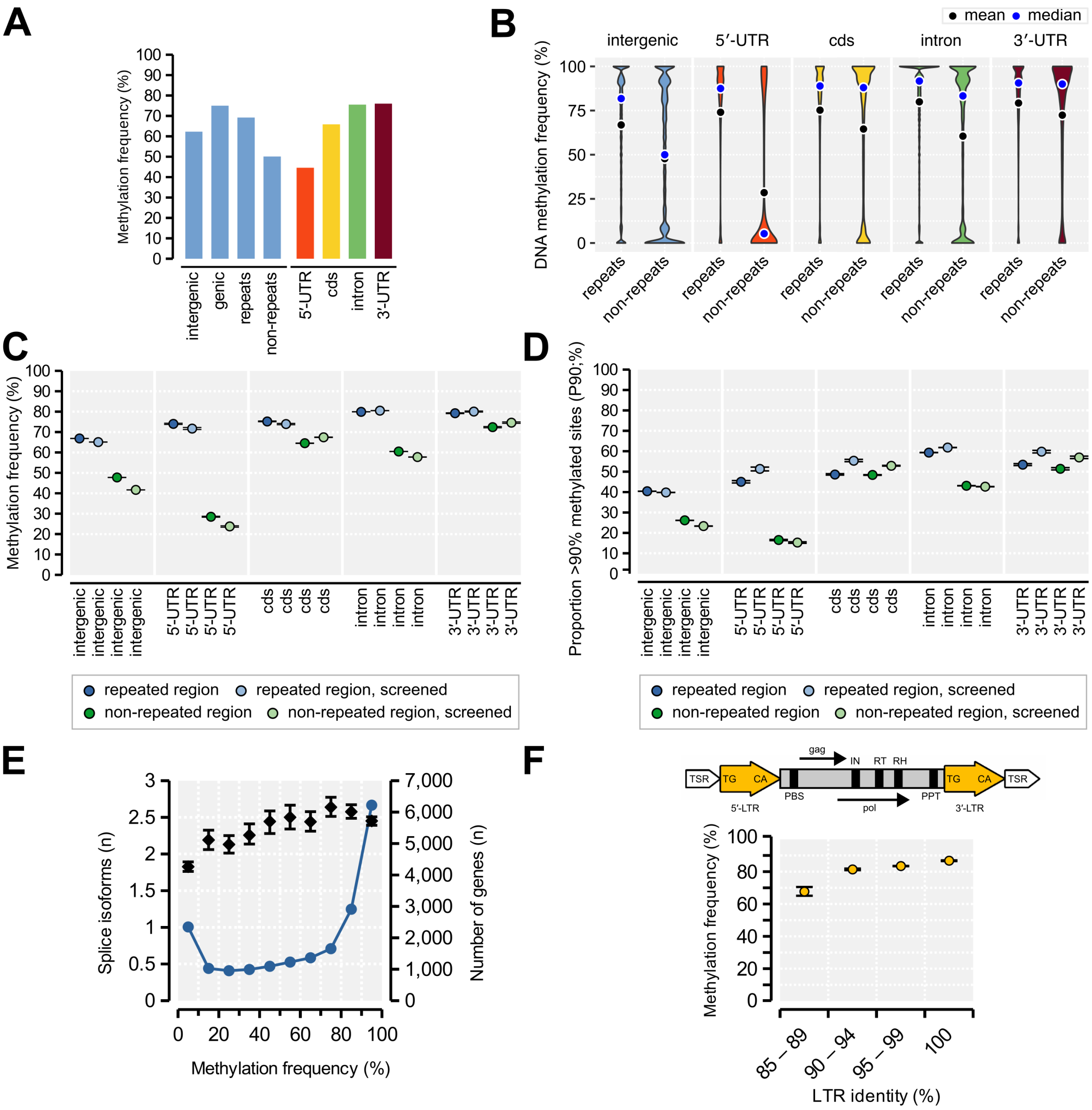


**Supplementary Figure 17:** Cytosine methylation frequencies detected at CpG sites through Nanopore signal analysis. (**A**) Overall DNA methylation frequencies (proportion of methylated Nanopore reads) in genic vs. intergenic regions, repeated vs. non-repeated regions and gene-body regions. Mean DNA methylation frequencies (f) and number of CpG sites (n): intergenic (f=62.3; n=61,170,962) genic (f=75.0; n=13,719,271); repeats (f=69.2; n=56,713,267); non-repeats (f=50.2; n=18,176,966); 5′-UTR (f=44.6; n=84,016); cds (f=65.9; n=478,953); intron (f=75.5; n=13,067,764); 3′-UTR (f=76.0; n=88,538). (**B**) Full distributions of DNA methylation rates across repeated vs. non-repeated regions, partitioned by intergenic and genic regions. Number of CpG sites (left to right): n=46,469,316; n=14,701,646; n=29,680; n=54,336; n=59,858; n=419,095; n=10,107,603; n=2,960,161; n=46,810; n=41,728. (**C**) Mean DNA methylation frequencies across intergenic and genic gene regions. “Screened” categories indicate measurement only across CpG sites that have been screened to exclude: i) heterozygous genotypes in the reference specimen; ii) regions occurring outside of the approved read-depth boundaries in the population genomic dataset. Whiskers indicate 95% confidence intervals generated from 200 non-parametric bootstrap pseudo-replicates (two-tailed comparisons). Number of CpG sites (left to right):

n=46,469,316; n=24,437,129; n=14,701,646; n=8,582,394; n=29,680; n=12,757; n=54,336; n=38,083; n=59,858; n=26,727; n=419,095; n=351,842; n=10,107,603; n=6,459,909; n=2,960,161; n=2,064,853; n=46,810; n=26,950; n=41,728; n=31,161 (**D**) As (C) but showing P90 values. P90 is the proportion of CpG sites with >90% methylated reads. Number of CpG sites as in (C). (**E**) The average number of RNA splice isoforms per gene as a function of average methylation rate in exons binned for intervals of 10% (y1-axis). Whiskers indicate 95% confidence intervals generated from 1000 non-parametric bootstrap pseudo-replicates (two-tailed comparisons). Number of genes in each bin shown on the y2-axis. Only gene models supported by RNA-seq data and with at least one CpG site with methylation data were included in the analysis (n=19,777 genes). (**F**) Average CpG-methylation rate across 1,706 putative LTR retrotransposons detected with LTR_Retriever and tested to contain at least one expected LTR domain. The model of a retrotransposon (top) indicates the location of the 5'-LTR and 3'-LTR regions (yellow; modified after a model in the LTR_Finder program manual) that were measured for identity for each element and used to bin elements into intervals of 5% divergence (low divergence between LTRs represent evolutionarily young repeats that may recently have been inserted into their respective genomic locations). Whiskers indicate 95% confidence intervals generated from 200 bootstrap replicates (two-tailed comparisons). The group of retrotransposons with 100% identical LTRs were found to have 1.28× times higher than those with 85–90% LTR identities (87% vs. 68%; $p<0.05$). Number of repeats per identity interval (left to right): n=7; n=91; n=1397; n=211. Number of CpG sites per identity interval (left to right): n=549; n=6,008; n=110,085; n=14,021.

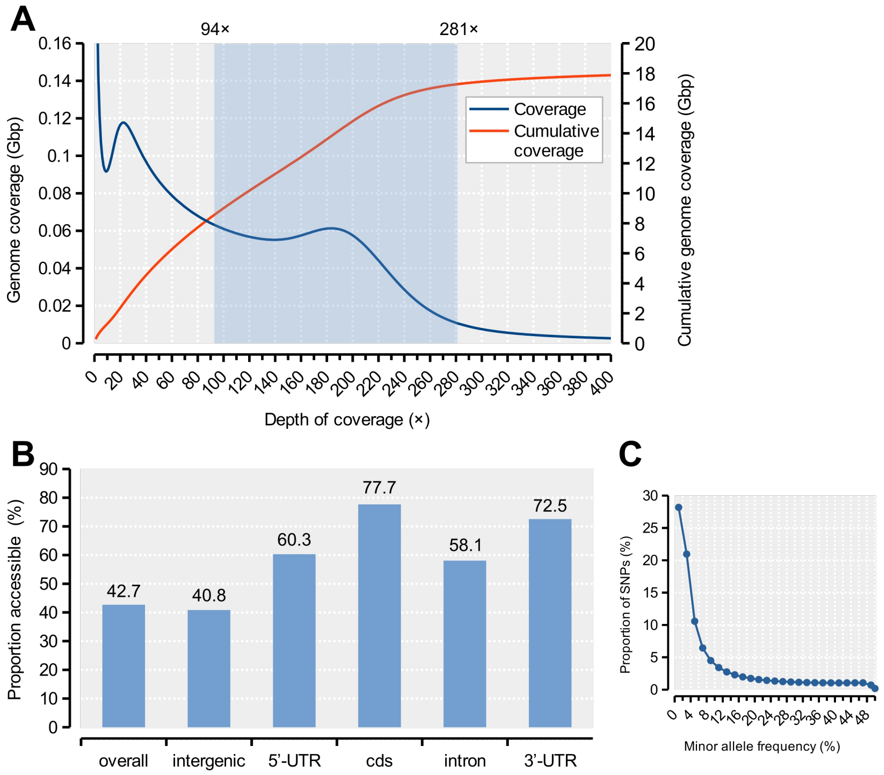


**Supplementary Figure 18:** Regions of the genome used to estimate levels and patterns of variation from SNPs. (**A**) A putative diploid peak of mapped short-read resequencing data was identified at ~188× depth of coverage (with minimum mapping qualities at 10 or more). Upper and lower thresholds at +50% (281×) and -50% (94×) around the peak were used to define the region of the genome accessible for analysis of variation (blue area). (**B**) In addition to the threshold in (A), a second criterion of at least 50% of samples being genotyped at a site was applied. The bars indicate the proportion of accessible sites in different genome regions given the thresholds specified in (A) and the minimum genotyping rate, spanning 8.4 Gb in total. Total number of positions per genomic region, including inaccessible sites (n): overall=19,733,156,487; intergenic=17,680,452,768; 5′-UTR=7,608,202; cds=44,154,034; intron=1,985,092,395; 3′-UTR=15,825,945. (**C**) The resulting folded allele frequency spectrum of all 760 million SNPs after applying the quality, depth and sample coverage filters.

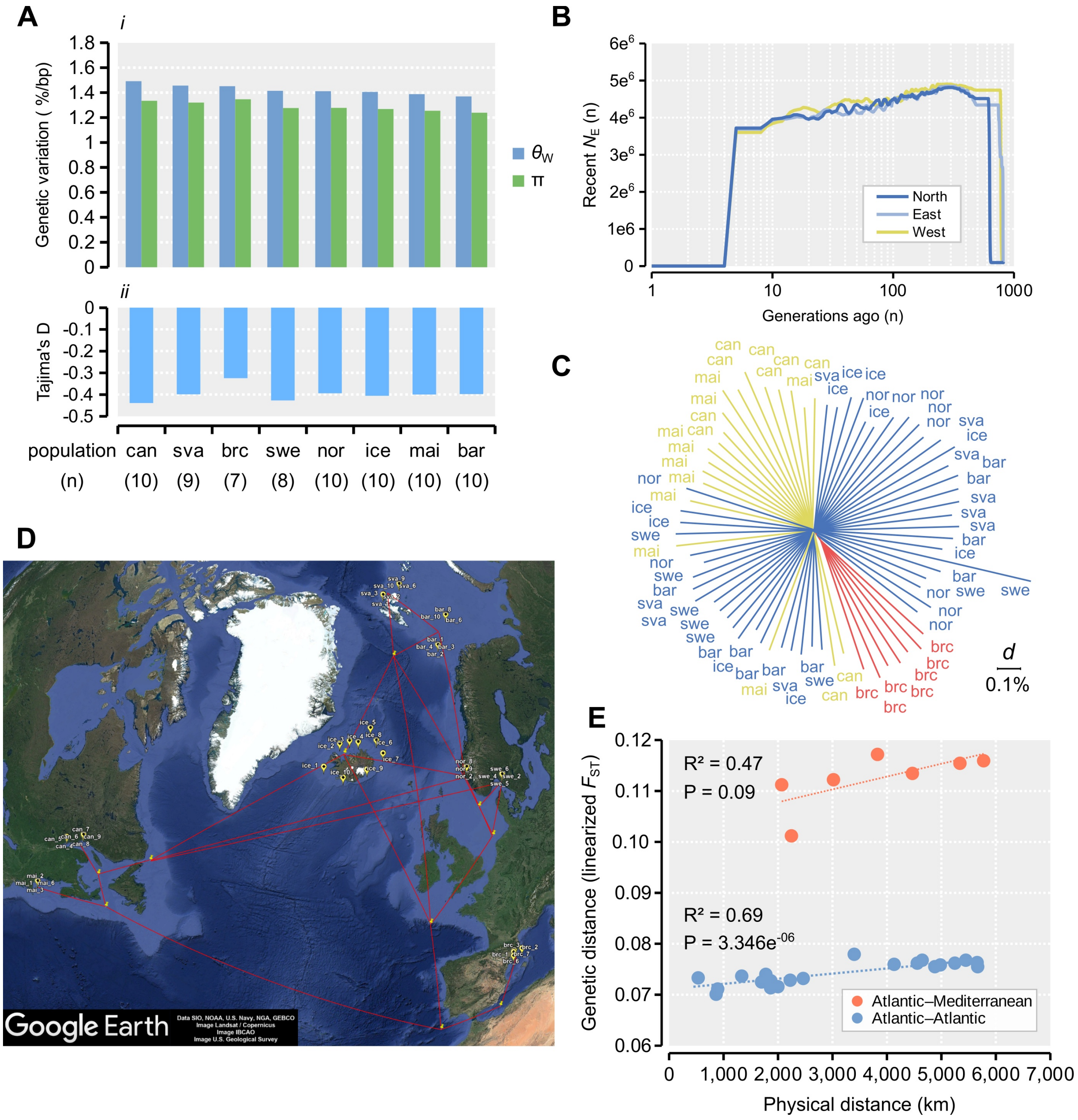


**Supplementary Figure 19:** Levels of genetic variation and genetic distances among populations (n=8) and samples (n=74). (**A**) *i*. Genome-wide mean levels of genetic variation in each population estimated using the population mutation rate ($\theta_W$) and nucleotide diversity ($\pi$) (can=Canada/Gulf of St. Lawrence; mai=USA/Gulf of Maine; ice=Iceland; sva=Svalbard; bar=Barents Sea; nor=Norway/Masfjord; swe=Sweden/Gullmarsfjord; brc=Spain/Catalan Sea). *ii*. Tajima's D inferred from $\theta_W$ and $\pi$ for the same populations. Sample sizes are given in parenthesis. (**B**) Inference of effective population size in the recent past from the spectrum of linkage disequilibrium among SNPs along the 199 longest scaffolds (247 Mb) in three regional gene pools: North=sva+bar (n=19); East=nor+swe (n=18); West=can+mai (n=20). (**C**) Neighbor-joining tree computed from genome-average per-base pairwise genetic distances (*d*) between all specimens (labels as in A) (n=760 M SNPs). (**D**) Physical distance vectors among populations and ocean regions, including mid- or branchpoints. (**E**) Genetic distance compared to physical distance. Linearized $F_{ST}$ was computed between all pairs of populations and matched with their corresponding shortest physical distance along the

vectors in (D) Dotted lines indicate linear regressions fitted to the distributions and $R^2$ values are the respective goodness of fit for each comparison. North Atlantic Ocean vs. Mediterranean Sea populations: n=7 pairs; F-statistic: 4.397 on 1 and 5 degrees of freedom, p-value: 0.09011. North Atlantic vs. North Atlantic: n=21 pairs; F-statistic: 41.88 on 1, 19 degrees of freedom, p-value: $3.346e^{-06}$. Seven individuals per population (Supplementary Data 8).

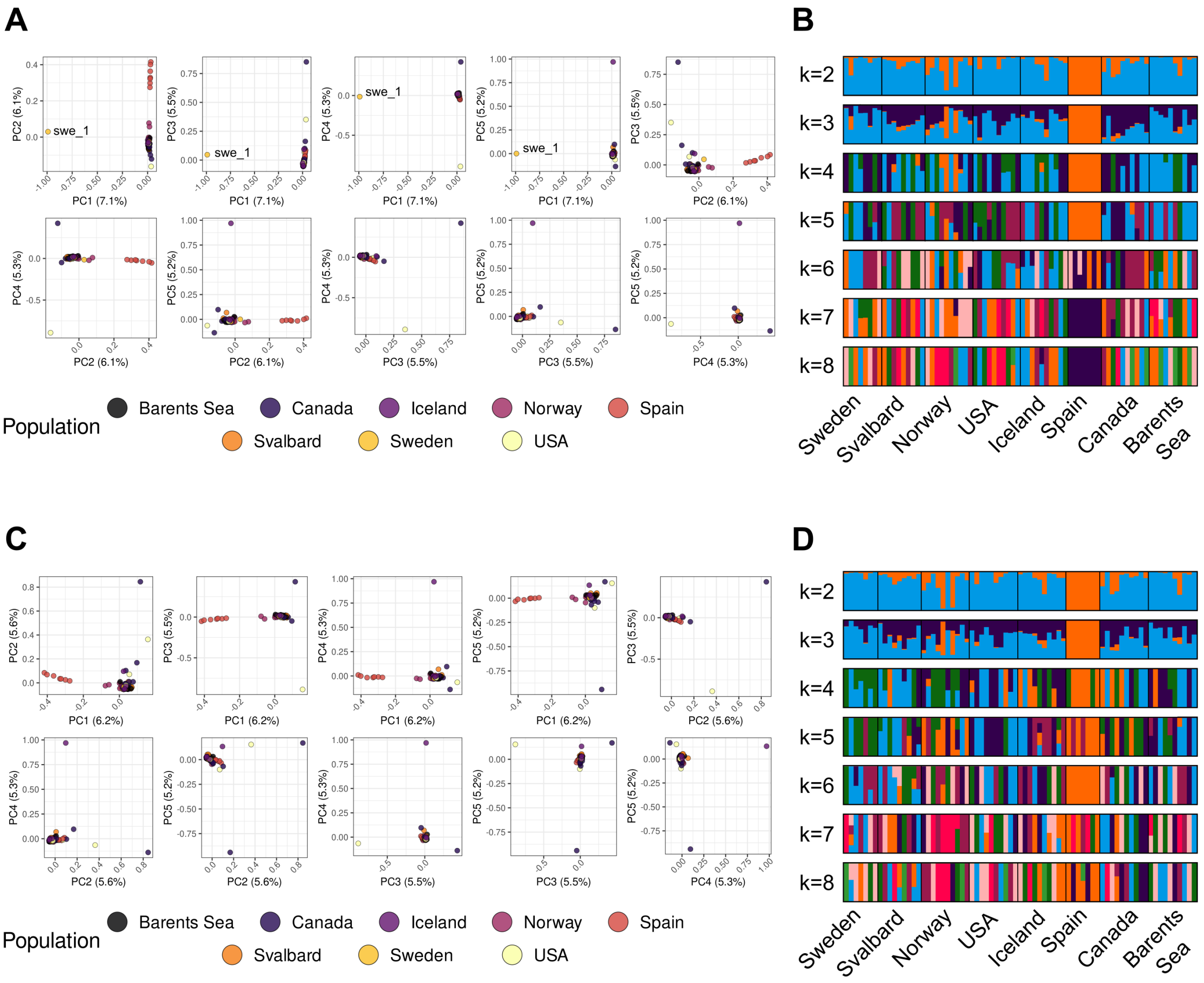


**Supplementary Figure 20:** Signatures of population structure and ancestry components from Principal Component Analysis (PCA) and ADMIXTURE analyses (n=7.35 M downsampled and unlinked SNPs). Top panels (A, B): results including the reference individual, which was sequenced to greater depth than the resequencing individuals. (**A**) The reference individual accounts for most of the variation in PC1, owing to an excess of called and private variants compared to the other individuals. PC2 shows some separation of the Spanish individuals. (**B**) ADMIXTURE results showing ancestry proportions for each individual across analyses assuming k=2–8 hypothetical ancestral populations (represented by different colors). The results suggest Spain as a separate population for k=2–5, but no clear substructure for remaining populations. Bottom panels (C, D): results excluding the reference individual. (**C**) Separation of Spanish individuals observed in PC1. Higher components show no clear population grouping. (**D**) ADMIXTURE results as in (B) suggesting Spain as a separate population for k=2–3, but no further substructure is observed.

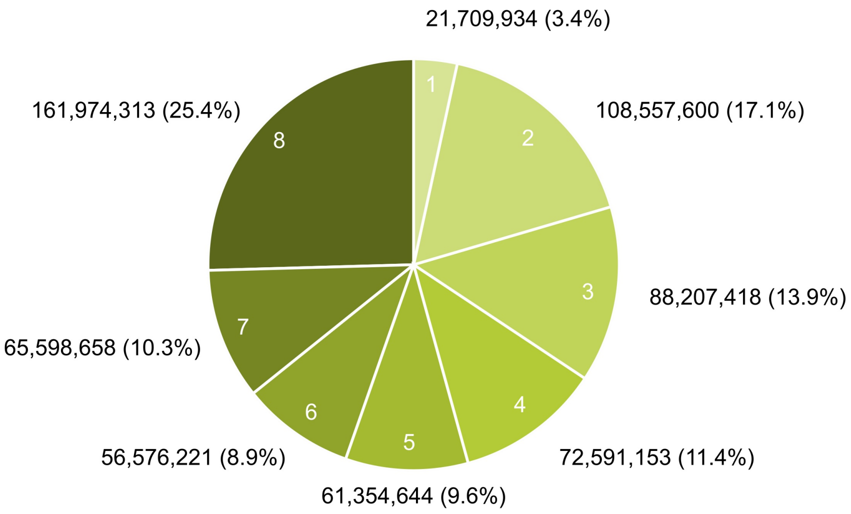


**Supplementary Figure 21:** Allele sharing across the eight studied Northern krill populations. Variants with allele counts of 2 or higher across samples were counted (i.e. excluding singleton alleles). Labels 1–8 indicate in how many populations a particular SNP is polymorphic (as counts or proportions of all SNPs). In total, 60% of the non-singleton variation is shared by five or more populations and only 3% is private.

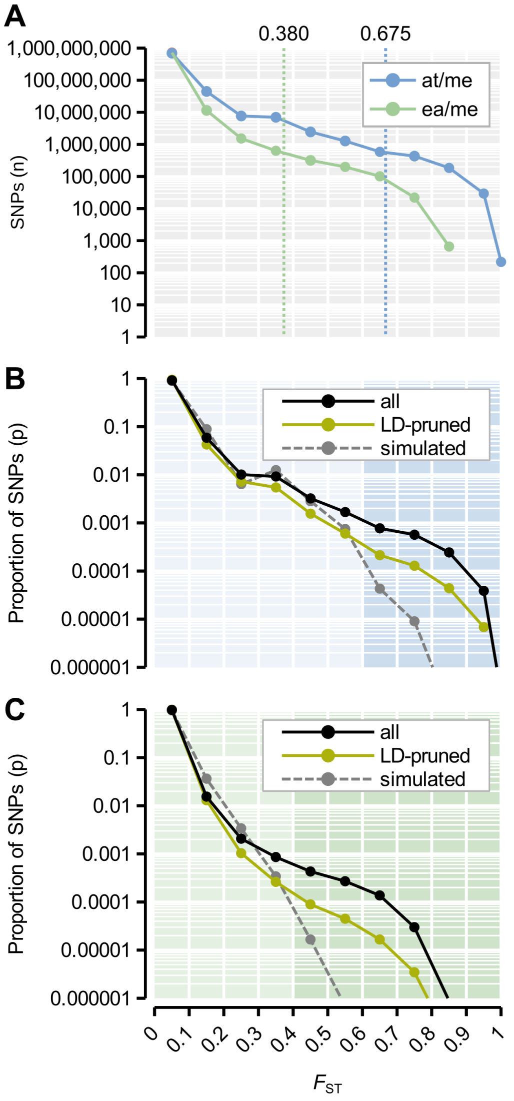


**Supplementary Figure 22:** Genetic divergence between krill sampled across the Atlantic Ocean and Mediterranean Sea. The two contrasts: i) "at/me"=North Atlantic Ocean samples (n=67) vs. the Mediterranean Sea samples (n=7); ii) "ea/we"=North Eastern samples from Iceland, the Barents Sea, Svalbard and Scandinavia (n=47) vs. South Western samples from Canada and the USA (n=20). (**A**) The numbers of SNPs at particular levels of allele frequency divergence in each contrast, as measured using the Fixation index $F_{ST}$ at each SNP. SNPs were binned in $F_{ST}$ intervals of 0.1. $F_{ST}$-scale goes from 0 to 1 as in (C). The top 0.1% percentiles for $F_{ST}$ are indicated for each contrast. (**B**) Observed vs. simulated divergence in the "at/me" contrast. Dotted lines indicate the proportion of SNPs at particular $F_{ST}$ levels for all data ("all"; n=760,436,665 SNPs), a subset of putatively independent SNPs selected to have low levels of linkage disequilibrium ("LD-pruned"; n=7,326,444 SNPs) and neutral SNPs simulated using the same overall divergence as the LD-pruned SNPs ($F_{ST}$=0.0561) under a simple scenario of population subdivision ("simulated; n=7,326,444 SNPs). SNPs were binned by $F_{ST}$ as in (A). For the shaded area of divergent SNPs ($F_{ST}$>0.6), the proportions (p) of "all" and "LD-pruned" observed SNPs are 31.1× and 7.5× higher than simulated SNPs ($p_{all}$=0.0016; $p_{LD\text{-}pruned}$=0.000393; $p_{simulated}$=5.21e$^{-05}$), respectively. (**C**) Observed vs. simulated divergence in the "ea/we" contrast using the same approach is in (B), comparing all data (n=738,107,753 SNPs), a subset SNPs with low levels of linkage disequilibrium (n=7,211,757 SNPs) and simulated SNPs (n=7,211,757 SNPs; overall

$F_{ST}$=0.0168). SNPs were binned by $F_{ST}$ as in (A). For the shaded area of divergent SNPs ($F_{ST}$>0.4), the proportions (p) of "all" and "LD-pruned" observed SNPs are 9.6× and 8.8× higher than simulated SNPs ($p_{all}$=0.00017; $p_{LD\text{-}pruned}$=0.00015; $p_{simulated}$=1.756e$^{-05}$), respectively.

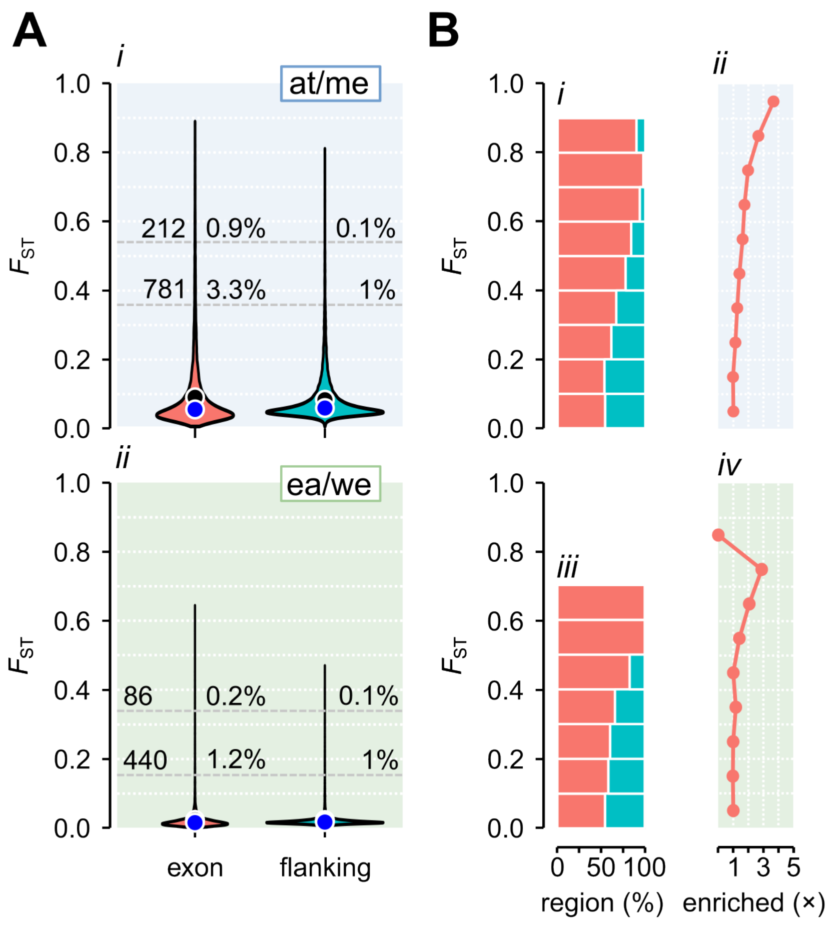


**Supplementary Figure 23:** Genetic divergence in exonic and flanking regions for the subset of ~25,000 functionally annotated genes. First contrast: "at/me"=North Atlantic Ocean samples (n=67) vs the Mediterranean Sea samples (n=7). Second contrast: "ea/we"=North Eastern samples from Iceland, the Barents Sea, Svalbard and Scandinavia (n=47) vs South Western samples from Canada and the USA (n=20). (**A**) The distribution of per-gene $F_{ST}$ values computed across the exons of genes (cds+UTRs) vs. flanking intergenic regions 50–100 kbp away from genes (circles: black=mean; blue=median) (*i*: n=23,554 vs. n=19,501; *ii*: n=23,542 vs. n=19,060). Gray lines are the 1% and 0.1% percentiles of flanking $F_{ST}$. The number and proportion of genes at that $F_{ST}$-level are indicated. (**B**) First contrast: (*i*) The relative proportion of genes vs. flanking regions at each level of $F_{ST}$. (*ii*) The enrichment of exonic vs. flanking windows (100 bp) at increasing divergence vs. undifferentiated regions ($F_{ST}$=0.0–0.1). Second contrast: *iii*–*iv* statistics as in *i*–*ii*.

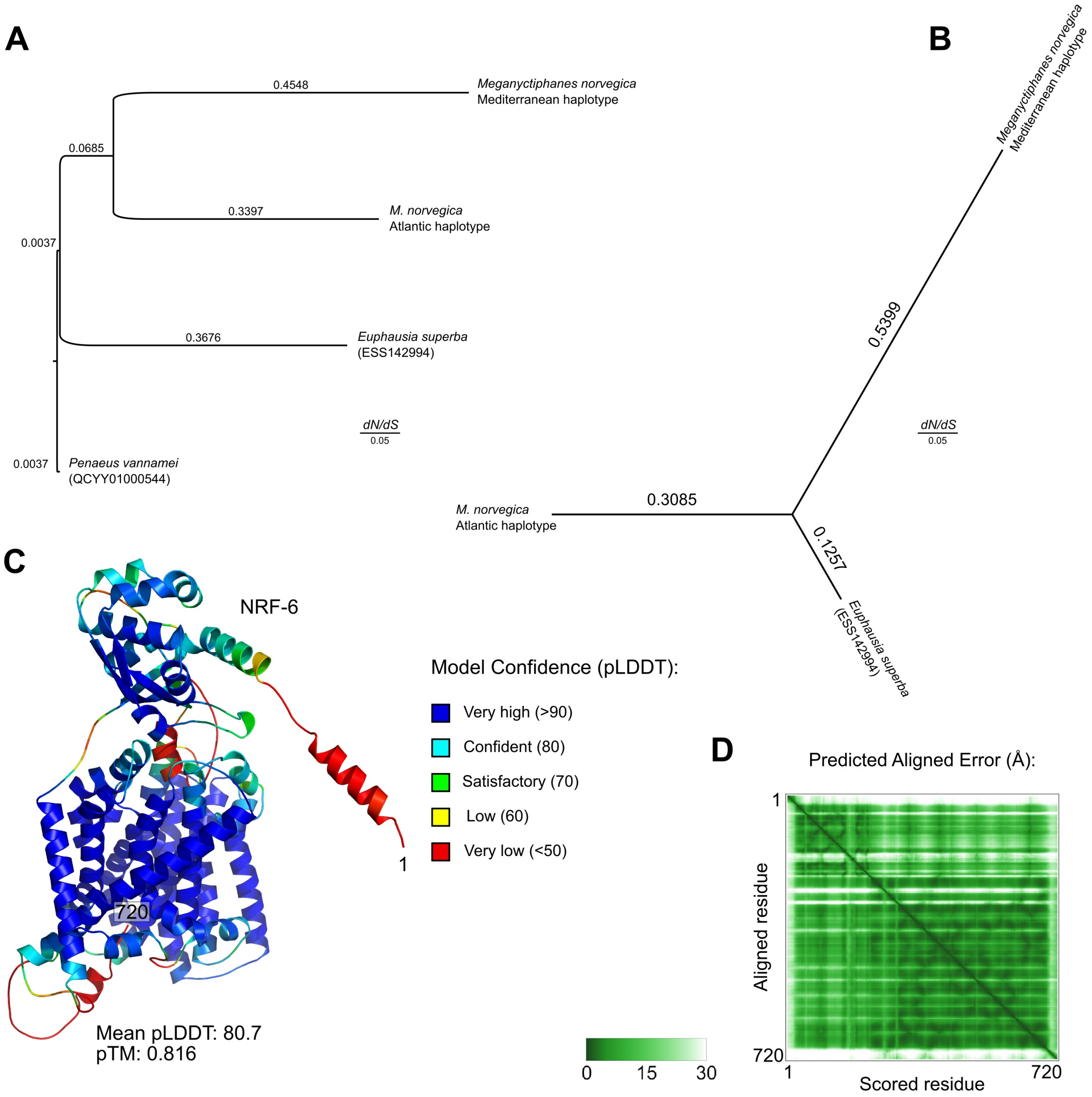


**Supplementary Figure 24:** Molecular evolution and topology of the *nrf-6* gene and encoded protein. (**A**) Maximum likelihood tree scaled by *dN/dS* along each branch (free-ratio model in PAML). The tree includes the Mediterranean and Atlantic Ocean haplotypes of the sequence in the Northern krill, as well as the homologous sequence in the Antarctic krill *Euphausia superba* and the shrimp *Penaeus vannamei*. (**B**). As in (A) but without the shrimp outgroup sequence. (**C**) Per-residue confidence coloring of the top ranked predicted model of NRF-6. The mean predicted local distance difference test (pLDDT) value and pTM score are annotated. (**D**) Residue-residue alignment plot of the predicted NRF-6 model.

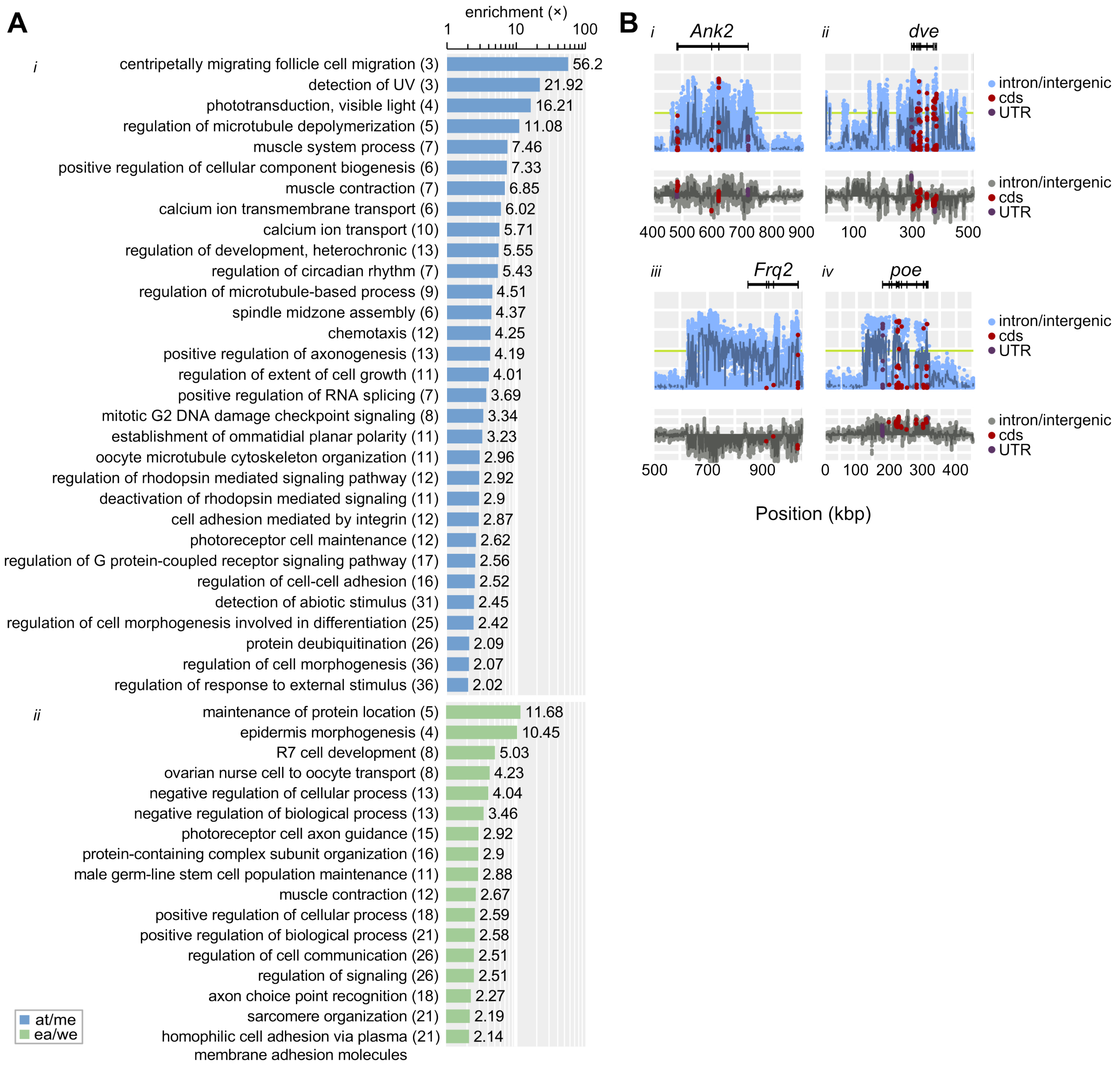


**Supplementary Figure 25:** Gene ontology enrichments (inferred with GOrilla) among divergent genes and examples of loci from the Atlantic contrast after removing small (<3 genes) or redundant ontologies (using Revigo). (**A**) For each of the two contrasts: *i*) at/me=Atlantic vs. Mediterranean krill; *ii*) ea/we=North-East North Atlantic vs South-West North Atlantic krill), the top enriched gene ontology terms from gene-lists ranked by exonic $F_{ST}$. Each list contains non-redundant terms summarized by Revigo after removal of enriched ontologies with few genes (less than three) or low enrichment compared to background (less than 2×). Number of genes associated with each term in parenthesis. (**B**) Examples of divergent genes in the North-East North Atlantic vs South-West North Atlantic contrast associated with regulation and signaling are homologs of fly genes: *i*) *Ankyrin 2* (*Ank2*; low identity score towards *Drosophila* homolog: BLAST e-value=0.75; *ii*) *defective proventriculus* (*dve*); *iii*) *Frequenin 2* (*Frq2*); *iv*) *purity of essence* (*poe*).

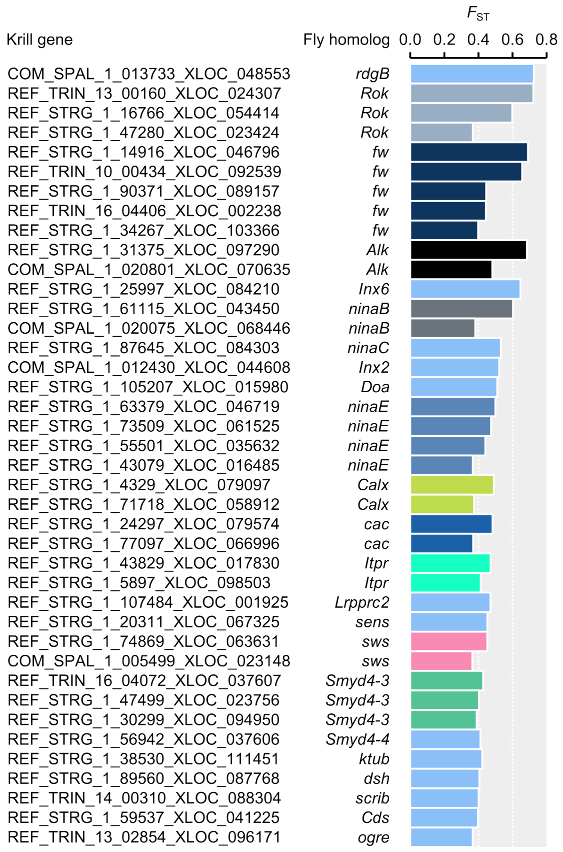


**Supplementary Figure 26:** Visual gene candidates for positive selection in the Atlantic vs. Mediterranean contrast (n=40) and their matching *Drosophila* homologs. Genes associated with significantly enriched eye functions (e.g. phototransduction, compound eye photoreceptor development, establishment of ommatidial planar polarity) were ranked by exon-wide $F_{ST}$ and grouped according to fly homolog. 27 out of 40 genes in this group of genes are putative gene family paralogs. See Supplementary Data 8 for additional homology and ontology details.

# Supplementary tables

## Supplementary Table 1.

Krill samples sequenced for genome assembly and population genetic analyses.

| Sample | Sequencing | Usage |
|---|---|---|
| K20 ("swe_1" in population dataset)<br>*M. norvegica* reference specimen | Long-read, linked-read and short-read DNA sequencing; RNA/cDNA long-read and short-read sequencing | Genome assembly, scaffolding, polishing and annotation; population genetic analyses |
| K4<br>*M. norvegica* specimen collected together with K20 | Long-read DNA trial sequencing | Preliminary mitochondrial draft assembly |
| Population dataset<br>74 *M. norvegica* specimens | Short-read sequencing | Depth-of-coverage estimation for haplotig identification in genome assembly; population genetic analyses |
| TI787-MN-M (adult male) | RNA short-reads (NCBI SRA: SRR3657321)[7] | RNA-based scaffolding and gene annotation |
| TI787-MN-F (adult female) | RNA short-reads (NCBI SRA: SRR3657320)[7] | RNA-based scaffolding and gene annotation |

**Supplementary Table 2.**

Statistics for the *M. norvegica* draft genome assembly and predicted genes.

| Feature | Count |
|---|---|
| Total length w/wo gaps (Gb) | 19.73 / 19.20 |
| Number of genome sequences (n) | 216,568 |
| Scaffold mean (bp) | 91,118 |
| Scaffold N50 / L50 (bp) | 220,856 / 24,545 |
| Scaffold N50 genic scaffolds (bp) | 421,132 |
| Scaffold N50 non-genic scaffolds (bp) | 132,640 |
| Scaffold N90 / L90 (bp) | 38,152 / 111,072 |
| Longest scaffold (bp) | 2,859,246 |
| Contig N50 (bp) | 46,549 |

**Supplementary Table 3.**

Gene models from other crustacean genome projects[8–14] used in SPALN for comparative gene detection.

| Species | Sequences (n) | Source |
|---|---|---|
| *Euphausia superba* (Antarctic krill) | 149,654 | Transcriptome; KrillDB |
| *Homarus americanus* (American lobster) | 40,732 | NCBI GCF_018991925.1 |
| *Penaeus monodon* (Black tiger shrimp) | 31,641 | https://www.biotec.or.th/pmonodon/index.php |
| *Penaeus vannamei* (Whiteleg shrimp) | 33,273 | NCBI GCF_003789085.1 |
| *Procambarus virginalis* (Marbled crayfish) | 22,206 | http://marmorkrebs.dkfz.de/ |
| *Cherax quadricarinatus* (Australian red claw crayfish) | 19,494 | https://www.frontiersin.org/articles/10.3389/fgene.2020.00201/full#supplementary-material |
| *Eriocheir sinensis* (Chinese mitten crab) | 7,549 | http://gigadb.org/dataset/100186 |

**Supplementary Table 4.**

Extra gene models from arthropod genome projects[8,12–15] used in EnTAP for functional gene annotation.

| Species | Sequences (n) | Source |
|---|---|---|
| *Homarus americanus* (American lobster) | 40,732 | NCBI GCF_018991925.1 |
| *Procambarus virginalis* (Marbled crayfish) | 22,206 | http://marmorkrebs.dkfz.de/ |
| *Cherax quadricarinatus* (Australian red claw crayfish) | 19,494 | https://www.frontiersin.org/articles/10.3389/fgene.2020.00201/full#supplementary-material |
| *Eriocheir sinensis* (Chinese mitten crab) | 7,549 | http://gigadb.org/dataset/100186 |
| *Parhyale hawaiensis* (amphipod) | 28,666 | https://research.janelia.org/pavlopoulos/ |
| *Daphnia pulex* (water flea) | 30,611 | ENSEMBL https://metazoa.ensembl.org/Daphnia_pulex/Info/Index |
| *Locusta migratoria* (migratory locust) | 29,872 | JAMg (536c4c1) i5k https://i5k.nal.usda.gov/locusta-migratoria |

**Supplementary Table 5.**

Crustacean species and published genome resources[8,9,11–13,16,17] used for orthology searches in ProteinOrtho and SwiftOrtho.

| Species | Genome size estimate (Gb) in publication | Non-redundant sequences (n) | 1:1 orthologues | Source |
|---|---|---|---|---|
| *Homarus americanus* (American lobster) | 3.06–4.64 | 22,355 | 7,150 | NCBI GCF_018991925.1 |
| *Cherax quadricarinatus* (Australian red claw crayfish) | 5 | 19,494 | 4,939 | https://www.frontiersin.org/articles/10.3389/fgene.2020.00201/full#supplementary-material |
| *Procambarus virginalis* (Marbled crayfish) | 3.5 | 21,773 | 4,027 | http://marmorkrebs.dkfz.de/ |
| *Penaeus monodon* (Black tiger shrimp) | 2.59 | 24,079 | 7,084 | https://www.biotec.or.th/pmonodon/index.php |
| *Penaeus vannamei* (Whiteleg shrimp) | 2.45 | 24,974 | 6,914 | NCBI GCF_003789085.1 |
| *Hyalella azteca* (amphipod) | 1.05 | 18,608 | 5,835 | NCBI GCF_000764305.1 |
| *Parhyale hawaiensis* (amphipod) | 3.6 | 28,666 | 6,093 | https://research.janelia.org/pavlopoulos/ |
| *Eurytemora affinis* (copepod) | 0.59–0.69 | 20,716 | 4,551 | NCBI GCF_000591075.1 |
| *Daphnia magna* | 0.15 | 16,878 | 4,533 | NCBI GCF_020631705.1 |

## Supplementary Note 1

### *No signature of whole-genome duplication (WGD) in the Northern krill*

To test for evidence of WGD in the krill, we investigated duplicated genes. The rate of gene family expansion is high compared to many crustaceans (Supplementary Fig. 8B; Supplementary Data 6), but there is no conclusive evidence from neutral divergence ($K_S$) among paralogs that many of them would have originated at the same time through WGD. Divergence-patterns are similar among all studied crustaceans. A shoulder in the $K_S$-distribution in krill is better modeled by multiple underlying distributions than one (Supplementary Fig. 8C–D; Supplementary Data 6), favoring smaller-scale duplications[18]. In vertebrates, horseshoe crabs and arachnids that have undergone WGD, many Hox gene ohnologs have been retained even after diploidization[19]. We found 9 out 10 core Hox genes (*lab*, *pb*, *Hox3*, *Dfd*, *Scr*, *ftz*, *Antp*, *Abd-A*, *Abd-B*, but not *Ubx;* Supplementary Fig. 11) in the krill genome but all but one are single-copy, not supporting WGD. Three putative *Hox3*-like paralogs were found. We therefore find that genome expansion in the krill is likely associated with TE proliferation and multiple small-scale duplications rather than WGD.

## Supplementary Note 2

### *Geographical variation in the Northern krill and regions used to sample material*

*M. norvegica* occurs over a wide geographic range (Fig. 1)[20]. Notable general physiological and behavioral characteristics and variations of the Northern krill across this range are that they: i) tend to grow larger and be more fecund in fjord-areas compared to pelagic areas[21]; ii) adjust to similar levels of oxygen consumption and metabolic rates across widely different prevailing ambient thermal conditions[20,22]; iii) initiate the reproductive season early (winter/spring) in the South and late (spring/summer) in the North to time spawning with phytoplankton blooms[23,24]; iv) tend to have a more carnivorous diet in the North and mixed omnivorous diet in the South[25]; and iv) prefer deeper depths and perform greater diurnal vertical migrations in oligotrophic (i.e. brighter) environments as a means to avoid predation[20,26,27].

### Gulf of Maine (USA) - The SW North Atlantic Ocean

The samples collected from the Gulf of Maine (USA) were part of the NSF OCE-1316040 grant and were, together with the Canadian samples, used to characterize the Northern krill from the South-Western range of its distribution. The Gulf of Maine is a large gulf, or inland sea, in the NE coast of the USA, bounded by Massachusetts, New Hampshire, Maine, and the Canadian provinces of New Brunswick and Nova Scotia by land, meanwhile in the ocean limits with Georges Bank and Browns Bank[28]. There are three major basins within the gulf (Jordan, Georges and Wilkinson Basins – the latter reaching below 250 m depth). At the Wilkinson Basin, where the samples were taken, surface water temperatures oscillate between 16 °C in the summer to about 7 °C in the winter. Below 50 m depth, water stays between 5 °C and 7 °C all year long[29,30]. Similar profiles are found in all the Gulf of Maine, although lower temperatures can be reached in areas under strong influence of the Labrador Sea water. Salinity ranges from 32.0 for those water masses of Scotian Shelf Water origin, to 34.6 in those typical from the Labrador Sea Water and 35.6 of the Warm Slope Water[30].

The Gulf of Maine is a high productivity area, with a strong spring bloom, summer surface stratification and fall mixing. Despite the important role and abundance of *M. norvegica* in the Gulf of Maine ecosystem, they show strong net avoidance due to their large size and swimming ability, therefore their true distribution, seasonality and long term-trends are not very well known[28,31].

### Gulf of Saint Lawrence (Canada) - The SW North Atlantic Ocean

The Gulf of Saint Lawrence samples were collected within the Atlantic Zone Monitoring Program (AZMP). The Gulf of Saint Lawrence is a semi-enclosed sea in South-East Canada. The deep Laurentian Channel runs from the continental shelf to the mouth of the Saint Lawrence River, surrounded by more shallow basins. In the winter, surface water temperature reaches below 0 °C, with temperature in depth ranging from below 0 °C (in shallow areas) to 8 °C in areas under the influence of slope waters entering the gulf, especially deep channels and basins. In contrast, during the summer, warmer waters are found at the surface (9 °C and warmer), while temperatures oscillate between 2 and 8 °C at greater depths[32]. Salinity is lower in the surface in summer, and in areas under the influence of the discharge of the Saint Lawrence river, meanwhile is relatively constant (about 34) in depth all year long[32].

In this region, *M. norvegica* forms loose aggregations in the deeper areas[33], showing feeding preference for copepods, especially *Calanus* spp., accumulating lipids during the fall and

early winter from the lipid-rich copepods in diapause, and consuming the reservoirs in spring and summer[34].

**Masfjord (Norway) - The NE North Atlantic Ocean**

The samples included in the study from Norwegian fjords were taken in Masfjorden, Nordhordaland on the second of October 2019 during a week-long cruise to threshold Fjords with the research vessel, Kristine Bonnevie. The Masfjord is a 24-kilometer long east-to-west directed fjord that empties into the inner part of the larger, and more open, Fensfjorden. It is between 500 to 1,500 meters wide, has a maximum depth of 494 m and the sill depth is 75 m, and is surrounded by relatively steep mountain sides. At the collection site (60°52.575909′ N 5°26.34410′ E), it is 421 meters deep and the samples were taken with a plankton net (MIK) at a depth between 202 and 306 meters. The temperature at the collection depth was measured to 8.5 °C and the salinity to 35.

*M. norvegica* is a dominant macroplankton in the Masfjord and particularly abundant in the head of the fjord, likely being advected there. They show a diurnal vertical migration pattern in the fjord, with most biomass concentrated in the upper 100 m during winter nights[35].

**Gullmar Fjord (Sweden) - The NE North Atlantic Ocean**

The Gullmar Fjord (sv. Gullmarsfjorden) is a narrow (1–2 km wide) sill fjord located on the Swedish west coast. It is 28 kilometers long and has a maximum depth of about 120 m, the basin with depths <100 m being about 5 kilometers long. The fjord is characterized by a unique combination of hydrographic conditions, being influenced by both the northward Baltic current, freshwater runoff and North Sea water. It has a brackish surface/upper layer (salinities between 24 and 27), an intermediate layer with salinities of 32–33 between 15–50 m and comparably stagnant deep layer with salinities 34–35 and temperatures that mostly fluctuate between 4–8 °C, and has suffered multiple low-oxygen events in the last 100 years[36]. The krill for this study was collected in the deep area, near Alsbäck-Fossen, in July 2018.

*M. norvegica* is the dominant euphausiid of the Gullmar Fjord and shows a diurnal vertical migration pattern with krill typically entering the upper/mid layers in the nighttime, and adults typically occurring at deeper depths than juveniles[37]. It was suggested this habitat is unusual in the sense that both beneficial access to nutrients and risk from predation are greater at deeper depths, reverse to most Northern krill habitats and that local vertical migration patterns reflect adjustment to local conditions[37]. The krill has high spawning and molting rates in the late summer in this area, followed by oosorption and reproductive diapause in the fall-winter[21].

**Iceland - The NE North Atlantic Ocean**

The samples from the Icelandic area were collected during the Icelandic spring cruise in May which is a monitoring survey where measurements are made on hydrography, nutrients, phytoplankton, and zooplankton around the island. The system of oceanic ridges on which Iceland rests divides the oceanic area around the island into different ocean regions or domains [38]. There is great variability in hydrographic conditions between these regions that in turn affect the distribution, composition and productivity of zooplankton [39–41]. To the south and west is the Atlantic Domain, characterized by relatively warm and saline Atlantic water with near surface winter temperatures (0-50 m) of ~6°C, and summer temperatures of ~12°C.

To the north is the Atlantic/Arctic Domain where the water is usually a mixture of Atlantic and Arctic water. Near surface temperatures are usually ~1°C during winter but may reach ~6°C during summer. To the east is the Arctic Domain with near surface temperatures usually below 0°C and may reach ~4–5°C during summer. The samples for this study were collected in all these three main hydrographic domains.

In Icelandic waters four euphausiid species are most abundant[42]. *Thysanoessa inermis* tends to be most abundant over the shelves, *M. norvegica* over the shelf edges, while *T. longicaudata* is mainly found in offshore areas. The fourth abundant euphausiid species, *T. raschi* is most common in fjords on the northwest, north and east coasts. In Icelandic waters, *M. norvegica* appears to have a life span of 2 years[43].

### Barents Sea & Svalbard - The NE North Atlantic Ocean

The high-latitude Barents Sea is characterized by a mix of warm Atlantic water (>3 °C), cold Arctic water (<0 °C) and warm coastal waters (>3 °C). The shelf area is about 1.6 million $km^2$ with a mean depth of 230 m, and the region is experiencing extensive warming and "Atlantification" through influx of Atlantic waters[44]. Krill make up a large part of the mesopelagic biomass and several *Thysanoessa* spp. are native to the region. *M. norvegica* is thought to be increasingly transported into the Barents Sea from the Norwegian Sea[45], and is also being advected into the Svalbard Archipelago and fjords[46,47]. The Arctic Ocean represents the northernmost range of the species and no evidence as of yet has been presented indicating it has formed resident reproducing populations in this area.

### Spain - The Mediterranean Sea

The Mediterranean sea is in general an oligotrophic area. It has constant temperature and salinity in the whole water column at all depths above 150–200 m and epipelagic water temperatures are between 13–28 °C in summer. This high temperature of the water column, in comparison to the neighboring Atlantic Ocean, may provoke a faster degradation of organic matter arriving to the Deep Sea bottom.

In the Mediterranean, *M. norvegica* has northern distribution. The lowest temperature where *M. norvegica* lives and completes its life cycle in the western Mediterranean is ~13 °C. Their highest densities in daytime are at depths >400–500 m*, though at night it occupies surface waters in the epipelagic zone[48,49], where also larvae (specially early stages) are mainly located[50]. There are some seasonal patterns in its density: in late winter–early spring, when the species breeds, it can aggregate inshore at the shelf-slope break[50,51]. In the fall, it moves deeper and can in autumn reach a second peak of abundance, downloading to 1000 m[51].

*M. norvegica* is the major prey of the Mediterranean fin whale[52], and it also occurs in the diet of deep-sea fish and shrimps, including near-bottom fish like juvenile *Merluccius merluccius* at the shelf-slope break[53]. At higher depths, to 1200 m, especially in autumn, a high variety of deep-sea bottom living fish (e.g. sharks) and crustaceans prey on it[54,55]. This has also been reported in Atlantic deep waters[56,57].

*as in the Bay of Cádiz, Atlantic Ocean.

## References


1. Urso, I. *et al.* A thorough annotation of the krill transcriptome offers new insights for the study of physiological processes. *Sci. Rep.* **12**, 11415 (2022).
2. Palecanda, S., Iwanicki, T., Steck, M. & Porter, M. L. Crustacean conundrums: a review of opsin diversity and evolution. *Philos. Trans. R. Soc. B Biol. Sci.* **377**, 20210289 (2022).
3. Zhang, Z.-M. *et al.* Crystal Structure of Human DNA Methyltransferase 1. *J. Mol. Biol.* **427**, 2520 (2015).
4. Dong, A. *et al.* Structure of human DNMT2, an enigmatic DNA methyltransferase homolog that displays denaturant-resistant binding to DNA. *Nucleic Acids Res.* **29**, 439–448 (2001).
5. Lu, L., Yi, C., Jian, X., Zheng, G. & He, C. Structure determination of DNA methylation lesions N 1 -meA and N 3 -meC in duplex DNA using a cross-linked protein–DNA system. *Nucleic Acids Res.* **38**, 4415–4425 (2010).
6. Hu, L. *et al.* Crystal Structure of TET2-DNA Complex: Insight into TET-Mediated 5mC Oxidation. *Cell* **155**, 1545–1555 (2013).
7. Blanco-Bercial, L. & Maas, A. E. A transcriptomic resource for the northern krill Meganyctiphanes norvegica based on a short-term temperature exposure experiment. *Mar. Genomics* (2017) doi:10.1016/j.margen.2017.05.013.
8. Polinski, J. M. *et al.* The American lobster genome reveals insights on longevity, neural, and immune adaptations. *Sci. Adv.* **7**, eabe8290 (2021).
9. Uengwetwanit, T. *et al.* A chromosome-level assembly of the black tiger shrimp (Penaeus monodon) genome facilitates the identification of growth-associated genes. *Mol. Ecol. Resour.* **21**, 1620–1640 (2021).
10. Sales, G. *et al.* KrillDB: A de novo transcriptome database for the Antarctic krill (Euphausia superba). *PLOS ONE* **12**, e0171908 (2017).
11. Zhang, X. *et al.* Penaeid shrimp genome provides insights into benthic adaptation and frequent molting. *Nat. Commun.* **10**, 1–14 (2019).
12. Gutekunst, J. *et al.* Clonal genome evolution and rapid invasive spread of the marbled crayfish. *Nat. Ecol. Evol.* **2**, 567–573 (2018).
13. Tan, M. H. *et al.* A Giant Genome for a Giant Crayfish (Cherax quadricarinatus) With Insights Into cox1 Pseudogenes in Decapod Genomes. *Front. Genet.* **11**, (2020).
14. Song, L. *et al.* Draft genome of the Chinese mitten crab, Eriocheir sinensis. *GigaScience* **5**, (2016).
15. Kao, D. *et al.* The genome of the crustacean Parhyale hawaiensis, a model for animal development,

regeneration, immunity and lignocellulose digestion. *eLife* **5**, e20062 (2016).

16. Poynton, H. C. *et al.* The Toxicogenome of Hyalella azteca: A Model for Sediment Ecotoxicology and Evolutionary Toxicology. *Environ. Sci. Technol.* **52**, 6009–6022 (2018).
17. Eyun, S. *et al.* Evolutionary History of Chemosensory-Related Gene Families across the Arthropoda. *Mol. Biol. Evol.* **34**, 1838–1862 (2017).
18. Vanneste, K., Van de Peer, Y. & Maere, S. Inference of Genome Duplications from Age Distributions Revisited. *Mol. Biol. Evol.* **30**, 177–190 (2013).
19. Schwager, E. E. *et al.* The house spider genome reveals an ancient whole-genome duplication during arachnid evolution. *BMC Biol.* **15**, 62 (2017).
20. Tarling, G. *Biology of Northern Krill*. (Academic Press, 2010).
21. Cuzin-Roudy, J., Tarling, G. A. & Strömberg, J.-O. Life cycle strategies of Northern krill (Meganyctiphanes norvegica) for regulating growth, moult, and reproductive activity in various environments: the case of fjordic populations. *ICES J. Mar. Sci. J. Cons.* **61**, 721–737 (2004).
22. Saborowski, R. & Buchholz, F. Metabolic properties of Northern krill, Meganyctiphanes norvegica, from different climatic zones: Enzyme characteristics and activities. *Mar. Biol.* **140**, 557–565 (2002).
23. CUZIN-ROUDY, J. Reproductive strategies of the Mediterranean krill, Meganyctiphanes norvegica and the Antarctic krill, Euphausia superba (Crustacea: Euphausiacea). *Invertebr. Reprod. Dev.* **23**, 105–114 (1993).
24. Cuzin-Roudy, J. Chapter Seven - Reproduction in Northern Krill (Meganyctiphanes norvegica Sars). in *Advances in Marine Biology* (ed. Tarling, G. A.) vol. 57 199–230 (Academic Press, 2010).
25. Mayzaud, P., Virtue, P. & Albessard, E. Seasonal variations in the lipid and fatty acid composition of the euphausiid Meganyctiphanes norvegica from the Ligurian Sea. *Mar. Ecol. Prog. Ser.* **186**, 199–210 (1999).
26. Tarling, G., Buchholz, F. & Matthews, J. The effect of lunar eclipse on the vertical migration behaviour of Meganyctiphanes norvegica (Crustacea: Euphausiacea) in the Ligurian Sea. *J. Plankton Res.* **21**, 1475–1488 (1999).
27. Onsrud, M. S. R. & Kaartvedt, S. Diel vertical migration of the krill Meganyctiphanes norvegica in relation to physical environment, food and predators. *Mar. Ecol. Prog. Ser.* **171**, 209–219 (1998).
28. Johnson, C. L. *et al.* Biodiversity and Ecosystem Function in the Gulf of Maine: Pattern and Role of Zooplankton and Pelagic Nekton. *PLOS ONE* **6**, e16491 (2011).
29. Du, J., Zhang, W. G. & Li, Y. Variability of Deep Water in Jordan Basin of the Gulf of Maine: Influence of Gulf Stream Warm Core Rings and the Nova Scotia Current. *J. Geophys. Res. Oceans* **126**,

e2020JC017136 (2021).

30. Townsend, D. W. *et al.* Water Masses and Nutrient Sources to the Gulf of Maine. *J. Mar. Res.* **73**, 93–122 (2015).
31. Lowe, M. R., Lawson, G. L. & Fogarty, M. J. Drivers of euphausiid distribution and abundance in the Northeast U.S. Shelf Large Marine Ecosystem. *ICES J. Mar. Sci.* **75**, 1280–1295 (2018).
32. Han, G., Loder, J. W. & Smith, P. C. Seasonal-Mean Hydrography and Circulation in the Gulf of St. Lawrence and on the Eastern Scotian and Southern Newfoundland Shelves. *J. Phys. Oceanogr.* **29**, 1279–1301 (1999).
33. McQuinn, I. H., Plourde, S., St. Pierre, J.-F. & Dion, M. Spatial and temporal variations in the abundance, distribution, and aggregation of krill (Thysanoessa raschii and Meganyctiphanes norvegica) in the lower estuary and Gulf of St. Lawrence. *Prog. Oceanogr.* **131**, 159–176 (2015).
34. Cabrol, J. *et al.* Trophic niche partitioning of dominant North-Atlantic krill species, Meganyctiphanes norvegica, Thysanoessa inermis, and T. raschii. *Limnol. Oceanogr.* **64**, 165–181 (2019).
35. Kaartvedt, S., Aksnes, D. L. & Aadnesen, A. Winter distribution of macroplankton and micronekton in Masfjorden, western Norway. *Mar. Ecol. Prog. Ser.* **45**, 45–55 (1988).
36. Filipsson, H. L. & Nordberg, K. Climate variations, an overlooked factor influencing the recent marine environment. An example from Gullmar Fjord, Sweden, illustrated by benthic foraminifera and hydrographic data. *Estuaries* **27**, 867–881 (2004).
37. Tarling, G. A., Cottier, F. R. & Everson, I. Spatial patterns in the vertical structure of euphausiids in Gullmarsfjord, Sweden: Identifying influences on bilayer formation and distribution. *Mar. Biol. Res.* **10**, 537–553 (2014).
38. Stefánsson, U. North Icelandic waters. *Rit Fiskid.* **3**, 1–269 (1962).
39. Gislason, A. Seasonal and spatial variability in egg production and biomass of Calanus finmarchicus around Iceland. *Mar. Ecol. Prog. Ser.* **286**, 177–192 (2005).
40. Malmberg, S.-A. & Valdimarsson, H. Hydrographic conditions in Icelandic waters, 1990-1999. *ICES J. Mar. Sci.* **219**, 50–60 (2003).
41. Valdimarsson, H., Astthorsson, O. S. & Palsson, J. Hydrographic variability in Icelandic waters during recent decades and related changes in distribution of some fish species. *ICES J. Mar. Sci.* **69**, 816–825 (2012).
42. Einarsson, H. Euphausiacea I. Northern Atlantic species. *Dana Rep* **27**, 1–185 (1945).

43. Silva, T., Gislason, A., Astthorsson, O. S. & Marteinsdóttir, G. Distribution, maturity and population structure of Meganyctiphanes norvegica and Thysanoessa inermis around Iceland in spring. *PLOS ONE* **12**, e0187360 (2017).

44. Eriksen, E., Rune Skjoldal, H., Gjøsæter, H. & Primicerio, R. Spatial and temporal changes in the Barents Sea pelagic compartment during the recent warming. *Prog. Oceanogr.* doi:10.1016/j.pocean.2016.12.009.

45. Eriksen, E. *et al.* The Barents Sea euphausiids: methodological aspects of monitoring and estimation of abundance and biomass. *ICES J. Mar. Sci. J. Cons.* **73**, 1533–1544 (2016).

46. Hünerlage, K. & Buchholz, F. Thermal limits of krill species from the high-Arctic Kongsfjord (Spitsbergen). *Mar. Ecol. Prog. Ser.* **535**, 89–98 (2015).

47. Dalpadado, P. *et al.* Distribution and abundance of euphausiids and pelagic amphipods in Kongsfjorden, Isfjorden and Rijpfjorden (Svalbard) and changes in their relative importance as key prey in a warming marine ecosystem. *Polar Biol.* **39**, 1765–1784 (2016).

48. Andersen, V., Sardou, J. & Nival, P. The diel migrations and vertical distributions of zooplankton and micronekton in the Northwestern Mediterranean Sea. 2. Siphonophores, hydromedusae and pyrosomids. *J. Plankton Res.* **14**, 1155–1169 (1992).

49. Sardou, J. & Andersen, V. Micronecton et macroplancton en Mer Ligue (Méditerranée) : migrations nycthémérales et distributions verticales. *Oceanol. Acta* **16**, 381–392 (1993).

50. Casanova, B. Les Euphausiacés de Méditerranée (Systématique et développement larvaire. Biogéographie et biologie). (Marseille, 1974).

51. Sardou, J., Etienne, M. & Andersen, V. Seasonal abundance and vertical distributions of macroplankton and micronekton in the Northwestern Mediterranean Sea. *Oceanol. Acta* **19**, 645–656 (1996).

52. Fossi, M. C. *et al.* Multi-trial biomarker approach in Meganyctiphanes norvegica: A potential early indicator of health status of the Mediterranean 'whale sanctuary'. *ResearchGate* **54**, 761–7 (2002).

53. Cartes, J. E., Hidalgo, M., Papiol, V., Massutí, E. & Moranta, J. Changes in the diet and feeding of the hake Merluccius merluccius at the shelf-break of the Balearic Islands: Influence of the mesopelagic-boundary community. *Deep Sea Res. Part Oceanogr. Res. Pap.* **56**, 344–365 (2009).

54. Carrasson, M., Stefanescu, C. & Carles, J. Diets and bathymetric distributions of two bathyal sharks of the Catalan deep sea (Western Mediterranean). *Mar. Ecol.-Prog. Ser. - MAR ECOL-PROGR SER* **82**, 21–30 (1992).

55. Cartes, J. E., Company, J. B. & Maynou, F. Deep-water decapod crustacean communities in the

Northwestern Mediterranean: influence of submarine canyons and season. *Mar. Biol.* **120**, 221–229 (1994).

56. Mauchline, J. & Gordon, J. D. M. Oceanic pelagic prey of benthopelagic fish in the benthic boundary layer of a marginal oceanic region. *Mar. Ecol. Prog. Ser.* **74**, 109–115 (1991).

57. Hudson, I. R. & Wigham, B. D. In situ observations of predatory feeding behaviour of the galatheid squat lobster Munida sarsi using a remotely operated vehicle. *J. Mar. Biol. Assoc. U. K.* **83**, 463–464 (2003).
