# Supplementary material for: Ecological genomics in the Northern krill uncovers loci for local adaptation across ocean basins
Source: Nat Commun. 2024 Aug 1;15:6297. doi: 10.1038/s41467-024-50239-7 (PMC11294593; doi:10.1038/s41467-024-50239-7)
Supplement: Supplementary file 4 — Description of Additional Supplementary Files [file 41467_2024_50239_MOESM4_ESM.pdf]

Description of Additional Supplementary Files

File Name: Supplementary Data 1

Description: Northern krill samples sequenced for genome assembly or population genomics.

File Name: Supplementary Data 2

Description: Sequence data produced to assemble and annotate the genome of the Northern krill.

File Name: Supplementary Data 3

Description: Repeats detected in the genome of the Northern krill.

File Name: Supplementary Data 4

Description: Gene annotations produced by TransDecoder and EnTAP.

File Name: Supplementary Data 5

Description: Comparative summary statistics of the lengths of genes and coding sequences and sequence divergence between the Northern krill and other crustacean species.

File Name: Supplementary Data 6

Description: Analyses of gene family evolution. CAFE analyses were not corrected for multiple testing. GO enrichment analyses were corrected for multiple testing.

File Name: Supplementary Data 7

Description: Datasets used in detecting hox genes or candidate genes for DNA methylation in the Northern krill.

File Name: Supplementary Data 8

Description: Levels of genetic diversity and divergence across the genome and genes

in the Northern krill.

File Name: Supplementary Data 9

Description: High F_{ST} variants (F_{ST}>0.5) in the coding region of nrf-6.

File Name: Supplementary Data 10

Description: : Gene ontology enrichment associated with putative adaptive divergence between populations and assessment of genes linked to photoreception. GO enrichment analyses were corrected for multiple testing.
